# Urban Governance, Multisectoral Action, and Civic Engagement for Population Health, Wellbeing, and Equity in Urban Settings: A Systematic Review

**DOI:** 10.3389/ijph.2023.1605772

**Published:** 2023-08-30

**Authors:** Cristina Mesa-Vieira, Nathalia Gonzalez-Jaramillo, Catalina Díaz-Ríos, Octavio Pano, Sophie Meyer, Marilyne Menassa, Beatrice Minder, Vivian Lin, Oscar H. Franco, Annika Frahsa

**Affiliations:** ^1^ Institute of Social and Preventive Medicine, University of Bern, Bern, Switzerland; ^2^ Graduate School for Health Sciences, University of Bern, Bern, Switzerland; ^3^ Public Health and Primary Care Library, University Library of Bern, University of Bern, Bern, Switzerland; ^4^ LKS Faculty of Medicine, University of Hong Kong, Hong Kong, Hong Kong SAR, China; ^5^ University Medical Center Utrecht, Utrecht, Netherlands

**Keywords:** equity, wellbeing, participatory health governance, multisectoral action, civic engagement

## Abstract

**Objectives:** To identify the validated and reliable indicators and tools to assess good governance for population health, wellbeing, and equity in urban settings, and assess processes of multisectoral action and civic engagement as reported by peer-reviewed articles.

**Methods:** We conducted a systematic review searching six databases for observational studies reporting strategies of either urban health, multisectoral action or civic engagement for wellbeing, health, or equity.

**Results:** Out of 8,154 studies initially identified we included 17. From the included studies, 14 presented information about high-income countries. The general population was the main target in most studies. Multisectoral action was the most frequently reported strategy (14 studies). Three studies used Urban Health Equity Assessment and Response Tool (Urban HEART). Health indicators were the most frequently represented (6 studies). Barriers and facilitators for the implementation of participatory health governance strategies were reported in 12 studies.

**Conclusion:** Data on the implementation of participatory health governance strategies has been mainly reported in high-income countries. Updated and reliable data, measured repeatedly, is needed to closely monitor these processes and further develop indicators to assess their impact on population health, wellbeing, and equity.

## Introduction

It has been estimated that by 2050 more than two-thirds of the world’s population will live in urban settlements (1). Intensive growth of cities leads to an increment of inequities and social exclusion, which can increase social, environmental, economic, and health risks (2). Therefore, to foster urban health, public policies that address social determinants are needed (3, 4). Being home to such a large proportion of the population, cities have been pointed out as important settings for implementing strategies that support the achievement of the Sustainable Development Goals (SDGs). The adoption of the SDGs in 2015 transformed the notion that urban policies are indeed health policies and provided a framework to promote urban health to close the gaps in health that emerge with urbanization (5). This framework has also contributed to the evidence of the links between the environment, health, and its determinants. By understanding these links, urban governance can be an approach that includes different stakeholders and dimensions to address health determinants and target several SDGs at once. Specifically, urban governance has been described as a mechanism to advance the knowledge of the processes that organize the relationship between different state agencies and civil society to make cities more inclusive and sustainable (6). It also determines how urban inequities and risks can be effectively tackled. Indeed, the same policies may have different impacts on different populations, however, this is because cities are complex systems. This variability in the effects can be attributed to the interactions between governance, stakeholders, and the civic population under forms of participatory governance aiming to create consensus on policymaking (7, 8). Furthermore, differences in the implementation strategies, particular contexts, and the indicators used for assessing performance could explain the heterogeneity. As urbanization trends continue, participatory urban governance (strategies that involve health governance, multisectoral action and civic engagement) gains relevance as a field of research.

Current evidence concerning participatory health governance, in the form of systematic reviews, has focused on the interventions or tools to assess the impact of the physical environment on urban health (9, 10). Other evidence (11, 12) has looked at the impact of intersectoral action on health equity. Chaparro et al reviewed assessment indicators of ‘healthy cities’ in Latin America and the Caribbean (13). Several indices, such as the global indicators framework for Sustainable Development Goals (14), the Social Progress Index (15), the universal Health Coverage Index (16), and the Indicators for Resilient Cities (17), are available to assess the progress on different dimensions and determinants that contribute to the achievement of the SGDs. However, there is scarce evidence in the scientific literature regarding the empirical applicability of these indices and indicators in urban settings. To date, there is scarce evidence that jointly examines participatory urban governance, civic engagement and multisectoral action, and that standardizes the available evidence on the indicators and tools to evaluate the impact of these strategies. Thus, our study aims to identify the validated and reliable indicators and tools to assess participatory governance for population health, wellbeing, and equity in urban settings, and assess processes of multisectoral action and civic engagement as reported by peer-reviewed articles.

## Methods

### Working Definitions

Urban governance: Good urban governance is defined as the process of interaction and decision-making to generate collective solutions through co-creation of practices and institutional engagement as part of a whole-of-government and whole-of society approaches (18).

Multisectoral action: A recognized relationship between part or parts of the health sector and part or parts of another sector, that has been formed to take action on an issue or to achieve health outcomes, (or intermediate health outcomes) in a way which is more effective, efficient or sustainable than could be achieved by the health sector working alone (19, 20).

Civic engagement: Involves the establishment of a new balance of rights and responsibilities and the redrawing of boundaries of state action and regulation. Engaged citizens are characterized as being politically, socially, and economically independent. Civic engagement aims to promote the quality of life in a community, through both political and non-political processes. It also includes forms of political, environmental, and community activism (21).

### Search Strategy

We conducted this systematic review according to PRISMA 2020 guidelines (22). We searched six databases (Medline (Ovid), Embase.com, Cochrane Library, Web of Science, Google Scholar, and Global Health (Ovid)) for observational studies reporting strategies of either urban health, multisectoral action or civic engagement for wellbeing, health or equity using a standardized tool published until 21 June 2021, and update until 14 June 2022. We developed a search strategy with the help of scientific information specialists and used a combination of search terms relating to urban settings (e.g., city, metropolitan area, and superblock), urban health governance (e.g., health policy), civic engagement (e.g., community participation/engagement, public involvement), multisectoral action (e.g., public-private partnership) and health, wellbeing, and equity. Duplicate records were removed using Deduklick (23). The search strategy is available in the Supplementary Section S1. We did not apply language restrictions. This study was registered in PROSPERO (CRD42021266564).

### Selection Criteria

We included observational studies reporting urban governance, multisectoral action or civic engagement strategies to achieve population health, equity, or wellbeing, measured with a standardized tool (e.g., survey, questionnaire, and interview) that report individual results from at least one city or superblock. We also included studies that followed a standardized process to identify or develop a set of indicators to assess participatory health governance strategies.

We excluded framework articles, protocols, letters to the editor, book chapters, systematic reviews and meta-analyses and non-peer-reviewed publications.

Five independent reviewers were trained on the topic, the inclusion and exclusion criteria, and the use of the data extraction software before starting the study. The first author screened all references and the other four a portion of all references, so all titles and abstracts were screened independently by two persons. We also performed the full-text screening in pairs. One person checked the eligibility of the study against the inclusion and exclusion criteria (Supplementary Section S2) and, if the article met all criteria, the reviewer extracted the data on a form that we previously designed on RedCap^©^ (24). The second reviewer then checked the eligibility and confirmed that the data was extracted accordingly. For the title and abstract, and the full-text screenings, both reviewers discussed disagreements and any unresolved disagreements were clarified with a third independent reviewer.

### Data Extraction and Synthesis

We extracted the data according to a protocol that we defined before the beginning of the study. These data included characteristics of the implemented strategy (type, date of implementation, policy domains), assessed outcomes, target population, and stakeholders. To characterize the urban settings in which the strategies took place, we classified the cities by size according to the Organisation for Economic Co-operation and Development parameters (OECD) (25). The classification depended on the number of inhabitants: large metropolitan area (1,500,000 inhabitants or more), metropolitan area (500,000–1,500,000 inhabitants), medium-size urban area (200,000–500,000 inhabitants), and small urban area (50,000–200,000 inhabitants). Furthermore, we classified the countries where the cities are located according to the World Bank income classification (26): low-, lower-middle-, upper-middle- and high-income. We also extracted data on the tools and indicators to assess the strategy, the prioritization of health equity gaps and gradients, and the barriers to implementation of the strategy.

For the data synthesis, we grouped the data into three categories. The first one collects the indicators identified for the evaluation of governance, multisectoral action and civic engagement processes. We then summarized the information about the impact of these strategies on different policy domains (health/healthcare, transportation, housing, sanitation, infrastructure, environment, education, economic conditions and social protection) to ultimately improve the population’s health, wellbeing, and equity. Finally, we condensed the available information on the barriers and facilitators for the implementation of participatory health governance. The data presented in the tables are described in the results section.

## Results

**FIGURE 1:**
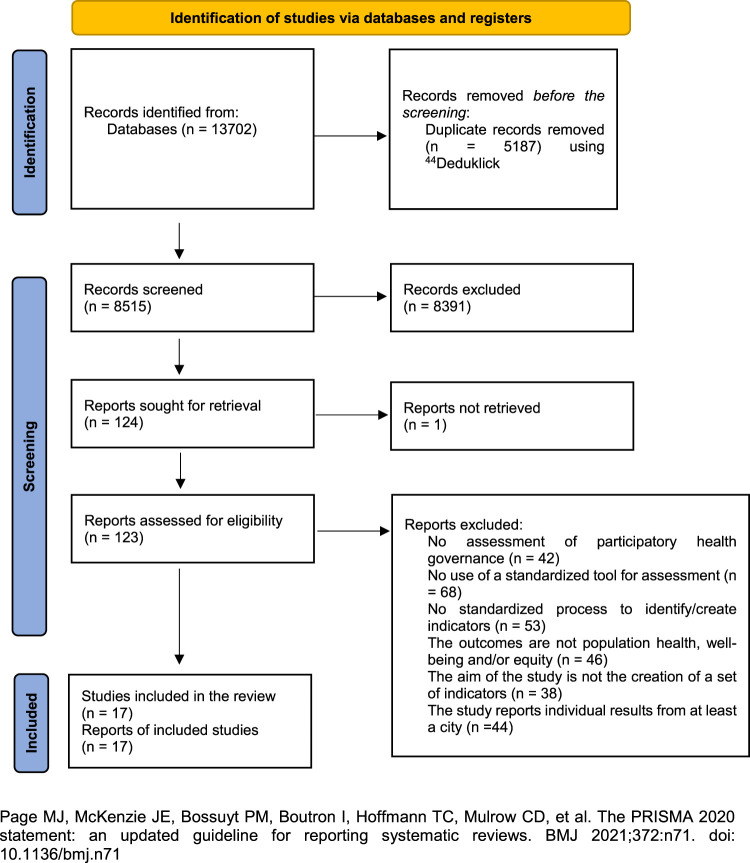
PRISMA (Preferred Reporting Items for Systematic Reviews and Meta-Analyses) 2020 flow diagram of the included studies (Switzerland, 2022).

Our search identified 8,516 studies. After assessing each study for eligibility, we included 17 independent studies (27–43) that met our inclusion criteria, as seen in the PRISMA flowchart (Figure 1).

**FIGURE 2:**
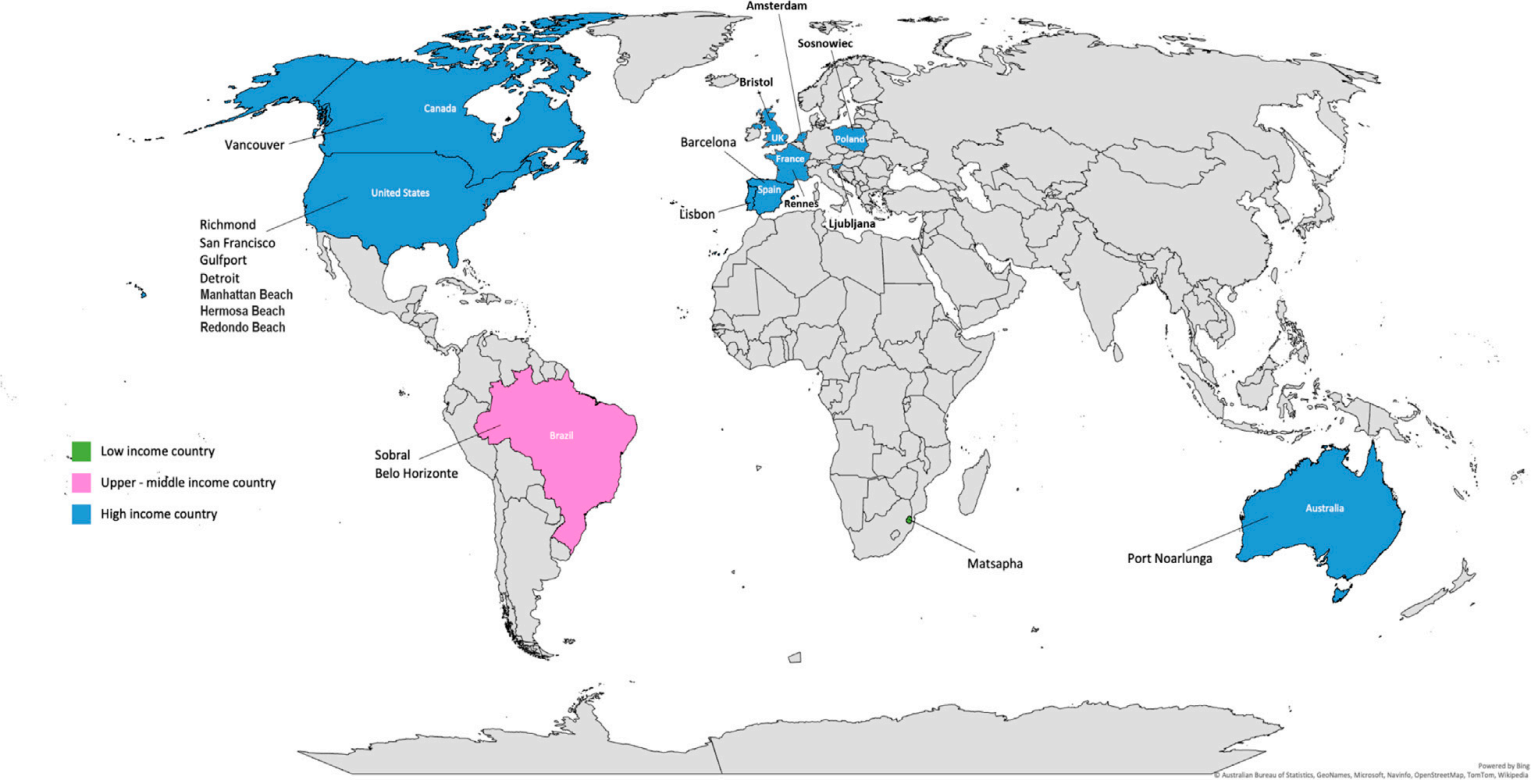
Geographic distribution of the included studies (Switzerland, 2022).

The majority of the studies (14) included in our systematic review presented information from cities located in high-income countries (28–37, 40–43), two from an upper-middle-income country (27, 38), and only one from a low-income country (39). As shown in Figure 2, high-income countries represented in the included studies are Spain, Canada, Australia, the United States, the United Kingdom, Netherlands, Slovenia, Poland, France and Portugal. Brazil was the only upper-middle-income country and Eswatini was the only low-income country. According to the OECD classification, five studies reported data from a large metropolitan area (28, 31, 37, 38, 41). Sobral, Richmond, Rennes, Bristol, Ljubljana and Sosnowiec were classified as medium-sized urban areas (27, 30, 32, 42); Barcelona, San Francisco, Lisbon, Detroit and Amsterdam were classified as metropolitan areas (33, 36, 40, 42); and Noarlunga, Gulfport, Hermosa Beach, Redondo Beach and Manhattan Beach as small-size urban areas (29, 34, 43). Matsapha and Vancouver were classified as other urban settings (35, 39).

The implementation of urban health governance, multisectoral action and civic engagement strategies, according to our search findings, peaked in the last decade. Only one study reported the implementation of such strategies in the 1980s (29), two studies between years 1990–1999 (27, 28), three studies between years 2000–2009 (31, 33, 37), and eleven reported strategies implemented between years 2010–2018 (28, 30, 32, 34–36, 39–43).

### Health Governance, Multisectoral Action and Civic Engagement

Multisectoral action was the strategy most frequently reported. As a lone process, multisectoral action to advance the construction of a healthy city was addressed in six (35%) studies (30–32, 35, 36, 41). Five studies assessed both multisectoral action and health governance (27, 29, 33, 38, 39); three reported data on multisectoral action and civic engagement (34, 37, 40), and the same amount of studies assessed only civic engagement (28, 42, 43).

The radar charts in Supplementary Figure S1 allow for a visual comparison of the proportion of the targeted population among the studies (Supplementary Figure S1A), the focus of the studies (Supplementary Figure S1B), and the dimensions of indicators that were used (Supplementary Figure S1C). The general population was the target in 14 studies (27, 29–33, 35–37, 39–43). Children were included in only three strategies (37, 38, 41) underserved populations in another three (30, 34, 41), youth in two (37, 41), ethnic groups in two (30, 34), and women (41), and the elderly (36) in one each. The impact of participatory health governance was observed on population health, equity and wellbeing in two of the studies (29, 34); on both population health and equity in another two (30, 39); and on population and wellbeing also in two (27, 33). The impact on population health and equity alone was looked at in four (28, 32, 35, 37) and six (31, 36, 38, 40–42) studies, respectively. Finally, one study looked at the impact of civic engagement on wellbeing (43).

Regarding the assessment of these strategies, only a few studies presented indicators to evaluate processes of participatory health governance. Barbieri (28), Corburn (30) and Fuertes (37) included indicators for governance, multisectoral action and civic engagement. Novoa (41) and Riley (43) contributed to our revision with indicators for civic engagement alone. Although multisectoral action was the most frequently reported strategy, more indicators to assess processes of health governance and civic engagement were included in the studies. Indicators to assess processes of participatory health governance, however, were only provided by strategies implemented in cities located in high-income countries (Richmond, Barcelona, Hermosa Beach, Manhattan Beach and Redondo Beach). The indicators that we identified in the selected studies are described in Table 1.

**TABLE 1:** Indicators to assess processes of health governance, civic engagement, and multisectoral action (Switzerland, 2022).

Indicators	Definition
Governance	
Health projects and community-based interventions implemented in the territory [N. Barbieri (28)]	This indicator specifies whether community-based health programs exist or not
The proportion of residents rating the value of services provided by the city as excellent or good [J. Corburn (30)]	Percentage of residents who rate the value of services provided by the city as excellent or good
The proportion of city employees who are women and/or minorities [J. Corburn (30)]	Percentage of city employees who are women and/or minorities
The proportion of residents reporting few or no experiences with racism and/or discrimination in the past year [J. Corburn (30)]	Percentage of residents who do not experienced racism or discrimination
Availability of neighbourhood health report, including quantitative and qualitative analysis of the current situation (yes/no) (%) [C. Fuertes (37)]	Coverage of the availability of neighbourhood health reports
Availability of an inventory of resources and current interventions in the neighbourhood (yes/no) (%) [C. Fuertes (37)]	Coverage of the availability of an inventory of resources and current interventions in the neighbourhood
Availability of a report providing a review of effective interventions to tackle the prioritized problems (yes/no) (%) [C. Fuertes (37)]	Coverage of the availability of a report providing a review of effective interventions to tackle the prioritized problems
Availability of a report with the action plan, objectives and interventions addressed to cover them (yes/no) (%) [C. Fuertes (37)]	Coverage of the availability of reports providing an action plan, objectives and interventions addressed to cover them
Percentage of interventions that cover the population envisaged [C. Fuertes (37)]	Percentage of interventions that cover the population envisaged
Percentage of interventions with an evaluation report [C. Fuertes (37)]	Percentage of interventions with an evaluation report
Civic engagement	
Existence of a community team that jointly approaches work with public resources and that works specifically on health (yes/no) [N. Barbieri (28)]	Existence of a community team that jointly approaches work with public resources and that works specifically on health
Existence of a neighbourhood health program [N. Barbieri (28)]	A neighbourhood health program forms part of a general rehabilitation policy that involves a strategy for community-based interventions in the field of health. It can also be seen as a program that inspires or strengthens other community-based health interventions
The proportion of residents that rate the job that the city does at involving citizens in policymaking for health, equity, and wellbeing as excellent or good [J. Corburn (30)]	Percentage of residents who rate the job that the city does at welcoming citizens in policymaking as excellent or good
The proportion of adults who volunteer on local boards, councils or organizations that address community problems [J. Corburn (30)]	Percentage of adults who participate in community service or volunteer work
Percentage of members very satisfied and absolutely satisfied (measured through Community Group Member Survey questionnaire) with the group progress and results, and community methodology [C. Fuertes (37)]	Percentage of members very satisfied and absolutely satisfied with the group progress and results, and community methodology
Availability of a report evaluating the satisfaction of the working group (yes/no) (%) [C. Fuertes (37)]	Coverage of the availability of a report evaluating the satisfaction of the working group
Availability of an ordered list of health problems, because of a participative prioritization workshop (yes/no) (%) [C. Fuertes (37)]	Coverage of the availability of an ordered list of health problems, because of a participative prioritization workshop
Existence of stable participatory structures for the implementation of community-based interventions [N. Barbieri (28)]	Existence of structures that establish links with the population and the public services of the territory to drive community-based interventions. Such structures have a multiplier effect on health promotion and illness prevention interventions that may be implemented in the territory
Percentage of interventions that have included an evaluation of participant’s satisfaction [C. Fuertes (37)]	Percentage of interventions that have included an evaluation of participant’s satisfaction
Voter abstention in the previous municipal elections (%) [A. M. Novoa (41)]	Percentage of eligible electorate who did not vote in the previous municipal elections
Awareness of the project in the city (yes/no) (%) [C. Riley (43)]	Percentage of residents that are aware of the existence of the project in their city
Level of engagement in the project (highly engaged/somewhat engaged/not at all engaged) (%) [C. Riley (43)]	Percentage of residents that assess their engagement with the project as either highly engaged, somewhat engaged or not at all engaged
The positive impact of the project on the resident’s life (strongly agree/agree/neither agree nor disagree/disagree/strongly disagree) (%) [C. Riley (43)]	The degree to which residents consider the project has impacted their life
The positive impact of the project on the community (strongly agree/agree/neither agree nor disagree/disagree/strongly disagree) (%) [C. Riley (43)]	The degree to which residents consider the project has impacted their community
Multisectoral action	
Participative prioritization, by the working group, of interventions to be implemented (yes/no) (%) [C. Fuertes (37)]	Percentage of interventions prioritized by the working group, based on the prioritization of detected problems, the review of effective interventions and the available resources and assets
The proportion of city contracts awarded to locally owned businesses [J. Corburn (30)]	Percentage of city contracts awarded to locally owned businesses
Percentage of links made with stakeholders envisaged: politicians, community professionals involved in health and social aspects (% coverage) [C. Fuertes (37)]	Percentage of links made with stakeholders envisaged: politicians, community professionals involved in health and social aspects
Establishment of a working group with stakeholders envisaged (% coverage) [C. Fuertes (37)]	Coverage of the establishment of a working group with stakeholders envisaged
Percentage of participants envisaged in the qualitative methods (professionals: sanitary, social, educational, community; representatives of neighbourhood entities, and citizens of both sexes, ages, and cultural origins) [C. Fuertes (37)]	Percentage of participants envisaged in the qualitative methods
Percentage of stakeholders envisaged who participate in the prioritization of health problems [C. Fuertes (37)]	Percentage of stakeholders envisaged who participate in the prioritization of health problems

### Impact of Participatory Health Governance on Different Policy Domains

In 13 of the studies included in the systematic review, a standardized tool, process, or index was used to assess participatory health governance. For this purpose, three (39–41) of these studies chose the Urban Health Equity Assessment and Response Tool (Urban HEART). Other tools and indexes were EuroQol, EnviroScreen, Urban Quality of Life Index, Life Evaluation Index (LEI), Cities Rapid Assessment Framework for Transformation (CRAFT) and the Population Health Index (Table 2). As represented in Supplementary Table S2, health indicators were reported in six studies (28, 29, 36, 38–40); housing (28, 32–34, 36, 40) and environmental indicators (28, 33, 34, 36, 40, 42) in five of them; indicators on transportation (28, 33, 36, 40, 43), education (27, 28, 36, 40), economic conditions and social protection in four (28, 33, 36, 40); and infrastructure in three (28, 33, 36). A smaller proportion of studies (two) presented indicators for sanitation (34, 39). In our synthesis, we found some common indicators used to assess the impact of different strategies in urban settings. Three studies reported using infant mortality rates as indicators for health and healthcare (27, 38, 39). More than one study also used the following: contraception use in persons 15–49 years (29, 39), fatality rates due to road traffic accidents (27, 36), and particulate matter concentrations (PM2.5 PM10) (36, 40, 42). Studies implemented in low-income countries provided indicators for health and sanitation only. Indicators on health, transportation and education are available from strategies implemented in Brazil (upper-middle-income); but indicators on housing, infrastructure, environment, economic conditions and social protection are provided from high-income countries only. Detailed information about the indicators according to diverse policy domains is available in Supplementary Table S2.

**TABLE 2:** Characteristics of the studies included in the systematic review (Switzerland, 2022).

Study	City	City size	Country	WB classification	Strategy implemented	Implementation date	Tool
Andrade et al (27)	Sobral	Medium-size urban area	Brazil	Upper-middle-income	Health governance, multisectoral action	1997–2002	N/A
Barbieri et al (28)	Barcelona	Large metropolitan area	Spain	High-income	Civic engagement	2014	Index of community action for health
Baum et al (29)	Noarlunga	Small urban area	Australia	High-income	Health governance, multisectoral action	1987–1990	N/A
Corburn et al (30)	Richmond	Medium-size urban area	United States	High-income	Multisectoral action	2012–2013	Cumulative toxic stressor model, EnviroScreen
Daban et al (31)	Barcelona	Large metropolitan area	Spain	High-income	Multisectoral action	2007–2018	N/A
Deloly et al (32)	Rennes	Medium-size urban area	France	High-income	Multisectoral action	2016-present	Cities Rapid Assessment Framework for Transformation (CRAFT)
Farhang et al (33)	San Francisco	Metropolitan area	United States	High-income	Health governance, multisectoral action	2004	Health impact assessment
Fastring et al (34)	Gulfport	Small urban area	United States	High-income	Multisectoral action, civic engagement	2016–2018	Policy maps, rankings, and roadmaps
Firth et al (35)	Vancouver, Victoria, Montreal, Saskatoon	Other	Canada	High-income	Multisectoral action	2016	Concept mapping
Freitas et al (36)	Lisbon	Metropolitan area	Portugal	High-income	Multisectoral action	2016–2017	Population Health Index
Fuertes et al (37)	Barcelona	Large metropolitan area	Spain	High-income	Multisectoral action, civic engagement	2007–2011	EuroQol
Junqueira et al (38)	Belo Horizonte	Large metropolitan area	Brazil	Upper-middle-income	Health governance, multisectoral action	1993–1997	Urban Quality of Life Index, Social Vulnerability Index
Makadzange et al (39)	Matsapha	Other	Eswatini	Lower-middle-income	Health governance, multisectoral action	2014	Urban HEART
Mehdipanah et al (40)	Detroit	Metropolitan area	United States	High-income	Multisectoral action, civic engagement	2016	Urban HEART
Novoa et al (41)	Barcelona	Large metropolitan area	Spain	High-income	Multisectoral action	2015	Urban HEART
Oliveira et al (42)	Bristol, Amsterdam*, Ljubljana, Sosnowiec	Medium-sized urban area, metropolitan area*	United Kingdom, Netherlands, Slovenia, Poland	High-income	Civic engagement	2015	N/A
Riley et al (43)	Hermosa Beach, Manhattan Beach, Redondo Beach	Small urban area	United States	High-income	Civic engagement	2010–2017	Life Evaluation Index (LEI)

WB, world bank; N/A, not available.

### Barriers and Facilitators for the Implementation of Participatory Health Governance

Barriers and facilitators for the implementation of participatory health governance strategies were reported in six of the studies (30, 31, 34, 40, 42, 43), and barriers alone were reported in another six of them (35–39, 41).

Common barriers were difficulties creating multisectoral working groups due to low citizen participation and motivation (31, 34, 35, 39), and the lack of data and analysis to assess the impact of the intervention, mostly at the neighbourhood level (34, 36–41). On the other hand, the authors expressed that the integration of different governmental entities (30, 40) and community training were facilitators for civic engagement in the development of healthy city strategies (31, 34). Oliveira (42) reported barriers associated with the gaps between the policymakers’ expectations and the investment and behavioural changes the community is willing to do. Therefore, they found that getting together to set priorities and dismissed unfeasible policies was a facilitator. In Riley’s study (43), they found that keeping track of outcomes and longitudinal monitoring was important to sustain policies throughout time while updating accordingly. Corburn (30) reported that the leadership of city authorities enabled the improvement of housing conditions, while Mehdipanah (40) reported that including the Urban HEART tool in the city’s surveillance system contributed to participatory health governance processes. Detailed information on barriers and facilitators is shown in Table 3.

**TABLE 3:** Barriers and facilitators for the implementation of participatory health governance (Switzerland, 2022).

Study	Barriers	Facilitators
J. Corburn (30)	• Structural racism and toxic stressors including environmental pollution, neighbourhood violence, unemployment, unsafe physical infrastructure and affordable access to quality goods and services, such as food, childcare, and healthcare, were barriers to being healthy	• Usage of integrative approach and structural racism lens
		• Usage of the power of eminent domain by the mayor and city council to support families under threat of losing their homes to foreclosure and to redevelop abandoned neighbourhoods
F. Daban (31)	• Limited citizen participation in the working groups	• The Public Health Agency of Barcelona provided training to community agents
	• The less active working group in health assessment due to lack of time or experience in methodological aspects	• The Catalonian Department of Health developed the COMSALUT program to facilitate methodology tools and coaching to primary healthcare teams and public health technicians to boost local community health
	• The difficulty of maintaining motivation over time	
	• Failure of action plan due to lack of communication, time, and conflicts of interests among the members of the working group	
	• The small sample size of participants per intervention	
D. Fastring (34)	• Due to limited citizen participation, their sample of participants was not representative of the neighbourhood as a whole	• The goals could be facilitated by engaging the community and continually seeking their input. The team is planning to offer Community Research Fellows Training to members of the neighbourhoods and residents in the city
	• Absence of demographic information at the city level	
C. L. Firth (35)	• Moderate participation rates given the lack of incentive, the accelerated timelines, and the profile of invited participants	N/A
A. Freitas (36)	• Limited data collection for indicators to assess urban health equity	N/A
C. Fuertes (37)	• Low quantitative information about the neighbourhoods	N/A
	• Poor information about the health impact of some interventions	
V. Junqueira (38)	• Limited data available	N/A
	• Limitations in the analysis of quantitative and qualitative data	
K. Makadzange (39)	• The long time needed to build an inclusive team	N/A
	• The long time needed to implement the tool	
	• Limited data were available	
R. Mehdipanah (40)	• Limited data were available	• Integration of the tool within the city’s health department could result in updated data from vital records and other sources
	• Available data with 1- or 2-year delay resulting in potentially outdated data	• Usage of mapping, as a technique for visualizing the distribution across geographic areas, to clarify the patterning of data
	• Usage of census tract- levels data can lead to challenges in interpreting and seeing patterns across many geographic areas	
A. M. Novoa (41)	• Estimation of indicators in areas with small populations	N/A
	• Difficulty in finding adequate physical context indicators at the neighbourhood level available periodically	
	• Data availability in the health domain	
K. Oliveira (42)	• Engagement of citizens with policies that entailed investment or behavioural changes	• Find common interests between policymakers and the community to set priorities and dismiss unreasonable policies
	• For policymakers, the costs of investing in new policies and achieving the implementation deadlines	
	• The gap between policymakers’ expectations and the willingness of the community to achieve the goals	
C. Riley (43)	• Recruitment and sustainability of community participants and leaders	• Adaptation of a methodology for assessing the impact of complex community-based interventions, tracking different outcomes longitudinally
	• Finding skilled community leaders and balancing their participation with instrumental stakeholders	
	• Implementing and sustaining multisector interventions	
	• Managing communication to sustain awareness, interest and involvement	
	• Managing the evolution of programs and campaigns to keep them “fresh” while maintaining fidelity	

## Discussion

### Main Findings

In this systematic review, we identified validated and reliable indicators and tools to assess participatory governance for population health, wellbeing, and equity in urban settings. We found that data on indicators to assess processes of participatory health governance and its impact on different policy domains is not frequently reported in the scientific literature. Moreover, most data on participatory health governance strategies comes from their implementation in metropolitan areas located in high-income countries while data from low- and middle-income countries is scarce. Most of the eligible studies were published in the last decade, demonstrating the growing interest and expansion of the field of participatory governance for health. We found that multisectoral action was the participatory strategy most frequently reported in studies. However, the majority of indicators evaluated processes of governance and civic engagement. We identified that only about half of the studies used standardized tools or developed indicators to assess the impact of participatory strategies, particularly on health, housing, and environmental issues. Citizen engagement and data constraints are the most mentioned barriers to implementing participatory health governance projects, while the leadership of city authorities and training of stakeholders were indicated as facilitators.

### Evidence in Context

Although we observed an expansion in the implementation of participatory health governance strategies in the last decade, efforts to build healthy cities have been underway before this time. In fact, in 1977, the World Health Organization (WHO) launched the “Health for All” initiative. This initiative aimed to achieve health and wellbeing by 2000 and called for a mobilization of resources to be invested in health, highlighting the role of multisectoral action and community involvement in doing so (44). Motivated by this initiative, the WHO Healthy network project was launched 30 years ago. Based on the premise that the living conditions, the economic situation and the physical environment have an impact on the health status of the population, this effort aims to put health in the agenda of decision-makers, promote a participatory approach to deal with the most relevant determinants of health in each city and create spaces to lobby for public health at the local level (45). Only in Europe, the Healthy Cities Network include more than 100 cities in 30 countries, but it extends to every WHO region (46). After the definition of the Millennium Development Goals (MDGs), the research on health governance started looking at the global level, underlining the role of international organizations in building capacity for participatory health governance. One of the main criticism about the MDGs has been the lack of a local perspective to empower citizens to improve the health status of their community (39). The shift to the Sustainable Development Goals (SDGs) represented an increase in the resources to support governance, since achieving SDGs required strong institutions and accountable governments with inclusive and participatory decision-making processes at all levels (47). However, it has been argued that bigger efforts are needed to foster health outside of the healthcare sector and put in place reformed strategies for multisectoral action that contribute to direct and indirect determinants of health beyond health systems (48).

As expected, we found differences in the type of indicators used to assess health, equity, and wellbeing according to the income classification of the country where cities were located. We did not find papers reporting on indices assessing the progress towards SDGs, such as the Social Progress Index (31) or the Global indicator framework for SDGs (14). Such indices might not have been used and reported at the city level since they are usually based on national or regional data that does not necessarily reflect the situation in urban settings.

Engaging multisectoral actors requires sufficient monetary resources to train stakeholders, organize the process of concertation and built the capacity to synthesize evidence relevant to policymaking. These resources are not always readily available in all settings (39). Notwithstanding, indicators to evaluate participatory governance can be used widely, regardless of the country or city’s income classification. Moreover, we believe that the availability of tools to assess models of urban governance and participatory strategies is particularly important for middle- and low-income countries where participatory governance may help to increase equity and enable more efficient use of resources allocated to benefit the most vulnerable groups and improve cities’ wellbeing (49). Besides multisectoral action, participatory urban governance benefits from civic engagement to achieve equitable policy development.

Civic engagement enables discussion of the city issues not only from the perspective of the government but also from the citizens, who can accurately express what they need and play an active role in prioritizing options and solutions. However, specific political and cultural contexts often interfere with the engagement of citizens during policy-making processes (44). Government systems are complex structures, and questions on who should participate, what level of knowledge is needed, and how to represent the interests of all population groups are frequently asked. Additionally, complex political structures, factors such as political instability, corruption (47), and resource constraints in low and middle-income settings, may also play a role as barriers to participatory governance processes. On the other hand, support from higher government levels and institutional infrastructure allowing the exchange of concerns and potential solutions may play an important role as facilitators for participatory health governance. For example, governmental authorities are motivated in creating and discussing the specific needs of smaller structures within a city, foster the design of targeted strategies and create a loop in which the results are regularly evaluated (44). Academic partners can contribute to the design and conduct research to inform and evaluate the progress of the strategies (48). Other stakeholders indirectly related to health, such as representatives of the housing, economy, and transportation sectors, are frequently underrepresented in practice and their impact on equity and wellbeing still needs further research (50). Beyond the general population, the perspective of particularly vulnerable groups such as ethnic minorities, older adults, and persons living with disabilities should be included as well. Underrepresented groups were rarely the target population of the included studies, and no indicators evaluated the perception of vulnerable groups. Such underrepresentation may have important implications as 1) policies and interventions could be designed and implemented without consideration for the priorities amongst these groups, and 2) priorities could remain unattended. Future studies could address how, across all levels of government and civil society, structures should be put in place for the elderly, children, women, and ethnic groups to be able to express their needs and to ensure their participation in policy design and implementation.

### Strengths and Limitations

To our knowledge, this is the first systematic review that synthesized evidence from studies reporting on participatory governance for health, equity, and wellbeing. However, this is a topic that is still developing, which is shown by the explosion of studies reporting participatory health governance in the last decade. The lack of research in the field limits the generalizability of our results. Moreover, as strategies for achieving healthy cities take years to impact population health, the data that has been published might not be up to date with the reality of the strategies for participatory action being currently implemented in several cities. Results from the implementation of participatory urban governance strategies is frequently not reported in the scientific literature. Publication of those initiatives should be encourage to improve the extant evidence and contribute to the development of strategies that benefit from the reported experiences of other cities. Although most information comes from the implementation in high-income countries, we did not restrict our search to geographical regions or income classification. This allowed us to compare the implementation of strategies for participatory governance in cities with different characteristics and present a set of indicators that can be adapted to the individual needs of each city. However, further complementary studies in more countries are needed to obtain a representative overview of the global situation. In addition to that, future research is needed, especially in the light of global developments in urban health and SDGs as well as processes and initiatives arising from the 2021 WHO Geneva Charter on Societal Wellbeing that empirically operationalizes those recent and comprehensive understandings of health and wellbeing.

### Conclusion

Our study shows that evidence on the implementation of participatory health governance strategies has been mostly published in high-income countries. Leadership from city officials and civic engagement are fundamental to fostering processes that help achieve the SDGs. Updated and reliable data is needed to closely monitor participatory processes and to assess their impact on the population’s health, wellbeing, and equity. Health, equity, and wellbeing indicators across cities differ greatly, and cities should prioritize needs and choose the indicators accordingly. However, indicators to evaluate participatory governance can be used widely, both in research and in implementing policies. Tools and indicators evaluating participatory processes may be helpful to evaluate the status of participation, follow up on the changes and analyze possible barriers and facilitators of those processes, particularly for underrepresented groups. Systematic evidence on participatory governance at the city level should be more widely available and the quality of the data should be strengthened.

## References

[1] Sinding SW. The Great Population Debates: How Relevant Are They for the 21st century? Am J Public Health (2000) 90(12):1841–5. 10.2105/ajph.90.12.1841 11111253 PMC1446443

[2] Hunter RF Cleland C Cleary A Droomers M Wheeler BW Sinnett D Environmental, Health, Wellbeing, Social and Equity Effects of Urban green Space Interventions: A Meta-Narrative Evidence Synthesis. Environ Int (2019) 130:104923. 10.1016/j.envint.2019.104923 31228780

[3] Marmot M Allen J Bell R Bloomer E Goldblatt P Consortium for the European Review of Social Determinants of Health and the Health Divide. WHO European Review of Social Determinants of Health and the Health divide. Lancet (2012) 380(9846):1011–29. 10.1016/S0140-6736(12)61228-8 22964159

[4] Navarro V Muntaner C Borrell C Benach J Quiroga A Rodriguez-Sanz M Politics and Health Outcomes. Lancet (2006) 368(9540):1033–7. 10.1016/S0140-6736(06)69341-0 16980120

[5] Ramirez-Rubio O Daher C Fanjul G Gascon M Mueller N Pajin L Urban Health: An Example of a “Health in All Policies” Approach in the Context of SDGs Implementation. Globalization and Health (2019) 15(1):87. 10.1186/s12992-019-0529-z 31856877 PMC6924052

[6] Raco M. Governance, Urban. In: Kobayashi A, editor. International Encyclopedia of Human Geography. 2nd ed. Oxford: Elsevier (2020). p. 253–8.

[7] Laverack G. Improving Health Outcomes through Community Empowerment: A Review of the Literature. J Health Popul Nutr (2006) 24(1):113–20. 16796158

[8] Zientara P Zamojska A Cirella GT. Participatory Urban Governance: Multilevel Study. PLoS One (2020) 15(2):e0229095. 10.1371/journal.pone.0229095 32084195 PMC7034860

[9] Pineo H Glonti K Rutter H Zimmermann N Wilkinson P Davies M. Urban Health Indicator Tools of the Physical Environment: A Systematic Review. J Urban Health (2018) 95(5):613–46. 10.1007/s11524-018-0228-8 29663118 PMC6181826

[10] Salgado M Vieira ACL Torres A Oliveira MD. Selecting Indicators to Monitor and Assess Environmental Health in a Portuguese Urban Setting: A Participatory Approach. Int J Environ Res Public Health (2020) 17(22):8597. 10.3390/ijerph17228597 33228088 PMC7699361

[11] Ndumbe-Eyoh S Moffatt H. Intersectoral Action for Health Equity: A Rapid Systematic Review. BMC Public Health (2013) 13:1056. 10.1186/1471-2458-13-1056 24209299 PMC3830502

[12] Shankardass K Solar O Murphy K Greaves L O'Campo P. A Scoping Review of Intersectoral Action for Health Equity Involving Governments. Int J Public Health (2012) 57(1):25–33. 10.1007/s00038-011-0302-4 21931976

[13] Chaparro R Melendi S Santero M Seijo M Elorriaga N Belizan M A Review of Assessment Indicators Used by Healthy Municipalities and Communities Program in Latin America and the Caribbean Region. Health Promot Int (2020) 35(4):714–29. 10.1093/heapro/daz059 31302691

[14] UNSTATS. Global Indicator Framework for the Sustainable Development Goals and Targets of the 2030 Agenda for Sustainable Development (2020).

[15] Social Progress Imperative. Social Progress Index. Washington, DC (2021).

[16] GBD 2019 Universal Health Coverage Collaborators. Measuring Universal Health Coverage Based on an index of Effective Coverage of Health Services in 204 Countries and Territories, 1990-2019: A Systematic Analysis for the Global Burden of Disease Study 2019. Lancet (2020) 396(10258):1250–84. 32861314 10.1016/S0140-6736(20)30750-9 PMC7562819

[17] Figueiredo L Honiden T Schumann A. Indicators for Resilient Cities (2018).

[18] Devas N. Urban Governance Voice and POverty in the Developing World. Routledge (2004).

[19] de Leeuw E. Intersectorality and Health: A Glossary. J Epidemiol Community Health (2022) 76(2):206–8. 10.1136/jech-2021-217647 34706927 PMC8761990

[20] Tangcharoensathien V Srisookwatana O Pinprateep P Posayanonda T Patcharanarumol W. Multisectoral Actions for Health: Challenges and Opportunities in Complex Policy Environments. Int J Health Pol Manag (2017) 6(7):359–63. 10.15171/ijhpm.2017.61 PMC5505105 28812831

[21] Ehrlich T. Civic Responsibility and Higher Education. American Council on Education/Oryx Press Series on Higher Education (2000).

[22] Page MJ McKenzie JE Bossuyt PM Boutron I Hoffmann TC Mulrow CD The PRISMA 2020 Statement: An Updated Guideline for Reporting Systematic Reviews. BMJ (2021) 372:n71. 10.1136/bmj.n71 33782057 PMC8005924

[23] Borissov N Haas Q Minder B Kopp-Heim D von Gernler M Janka H Reducing Systematic Review burden Using Deduklick: A Novel, Automated, Reliable, and Explainable Deduplication Algorithm to foster Medical Research. Syst Rev (2022) 11(1):172. 10.1186/s13643-022-02045-9 35978441 PMC9382798

[24] Harris PA Taylor R Thielke R Payne J Gonzalez N Conde JG. Research Electronic Data Capture (REDCap)-Aa Metadata-Driven Methodology and Workflow Process for Providing Translational Research Informatics Support. J Biomed Inform (2009) 42(2):377–81. 10.1016/j.jbi.2008.08.010 18929686 PMC2700030

[25] OECD. Urban Population by City Size (Indicator) (2022).

[26] World Bank. World Bank Country and Lending Groups 2021. Available at:https://datahelpdesk.worldbank.org/knowledgebase/articles/906519-world-bank-country-and-lending-groups.

[27] Andrade LO Bareta IC Gomes CF Canuto OM. Public Health Policies as Guides for Local Public Policies: The Experience of Sobral-Ceara, Brazil. Promot Educ (2005) 12:28–31. 10.1177/10253823050120030111x 16161846

[28] Barbieri N Gallego R Morales E Rodríguez-Sanz M Palència L Pasarín MI. Measuring and Analysing Community Action for Health: An Indicator-Based Typology and its Application to the Case of Barcelona. Soc Indicators Res (2018) 139:25–45. 10.1007/s11205-017-1703-4

[29] Baum F Cooke R. Healthy Cities Australia: The Evaluation of the Pilot Project in Noarlunga, South Australia. Health Promot Int (1992) 7(3):181–93. 10.1093/heapro/7.3.181

[30] Corburn J Curl S Arredondo G Malagon J. Health in All Urban Policy: City Services through the Prism of Health. J Urban Health (2014) 91(4):623–36. 10.1007/s11524-014-9886-3 25047156 PMC4134455

[31] Daban F Pasarin MI Borrell C Artazcoz L Perez A Fernandez A Barcelona Salut Als Barris: Twelve Years' Experience of Tackling Social Health Inequalities through Community-Based Interventions. Gac Sanit (2021) 35(3):282–8. 10.1016/j.gaceta.2020.02.007 32527681

[32] Deloly C Gall AR Moore G Bretelle L Milner J Mohajeri N Relationship-building Around a Policy Decision-Support Tool for Urban Health. Build Cities (2021) 2(1):717–33. 10.5334/bc.110 34704038 PMC7611888

[33] Farhang L Bhatia R Scully CC Corburn J Gaydos M Malekafzali S. Creating Tools for Healthy Development: Case Study of San Francisco's Eastern Neighborhoods Community Health Impact Assessment. J Public Health Manag Pract (2008) 14(3):255–65. 10.1097/01.PHH.0000316484.72759.7b 18408550

[34] Fastring D Mayfield-Johnson S Funchess T Egressy J Wilson G. Investing in Gulfport: Development of an Academic-Community Partnership to Address Health Disparities. Prog Community Health Partnersh (2018) 12(1S):81–91. 10.1353/cpr.2018.0023 29755051

[35] Firth CL Stephens ZP Cantinotti M Fuller D Kestens Y Winters M. Successes and Failures of Built Environment Interventions: Using Concept Mapping to Assess Stakeholder Perspectives in Four Canadian Cities. Soc Sci Med (2021) 268:113383. 10.1016/j.socscimed.2020.113383 32980679

[36] Freitas Â Rodrigues TC Santana P. Assessing Urban Health Inequities through a Multidimensional and Participatory Framework: Evidence from the EURO-HEALTHY Project. J Urban Health (2020) 97(6):857–75. 10.1007/s11524-020-00471-5 32860097 PMC7454139

[37] Fuertes C Pasarin MI Borrell C Artazcoz L Diez E Group of Health in the N. Feasibility of a Community Action Model Oriented to Reduce Inequalities in Health. Health Policy (2012) 107(2-3):289–95. 10.1016/j.healthpol.2012.06.001 22784994

[38] Junqueira V Pessoto UC Kayano J Nascimento PR Castro IEN Rocha JL Equidad en la salud: evaluación de políticas públicas en Belo Horizonte, Minas Gerais, Brasil, 1993-1997. Cad Saude Publica (2002) 18(4):1087–101. 10.1590/s0102-311x2002000400014 12118313

[39] Makadzange K Radebe Z Maseko N Lukhele V Masuku S Fakudze G Implementation of Urban Health Equity Assessment and Response Tool: A Case of Matsapha, Swaziland. J Urban Health (2018) 95(5):672–81. 10.1007/s11524-018-0241-y 29616450 PMC6181813

[40] Mehdipanah R Israel BA Richman A Allen A Rowe Z Gamboa C Urban HEART Detroit: The Application of a Health Equity Assessment Tool. J Urban Health (2021) 98(1):146–57. 10.1007/s11524-020-00503-0 33398612 PMC7781400

[41] Novoa AM Perez G Espelt A Echave C de Olalla PG Calvo MJ The Experience of Implementing Urban HEART Barcelona: A Tool for Action. J Urban Health (2018) 95(5):647–61. 10.1007/s11524-017-0194-6 29039133 PMC6181815

[42] Oliveira K Rodrigues V Slingerland S Vanherle K Soares J Rafael S Assessing the Impacts of Citizen-Led Policies on Emissions, Air Quality and Health. J Environ Manage (2022) 302:114047. 10.1016/j.jenvman.2021.114047 34741943

[43] Riley C Roy B Lam V Lawson K Nakano L Sun J Can a Collective-Impact Initiative Improve Well-Being in Three US Communities? Findings from a Prospective Repeated Cross-Sectional Study. BMJ Open (2021) 11(12):e048378. 10.1136/bmjopen-2020-048378 PMC8704973 34937711

[44] Goodwin E Ager A. Localisation in the Context of UK Government Engagement with the Humanitarian Reform Agenda. Front Polit Sci (2021) 3. 10.3389/fpos.2021.687063

[45] Goldstein G. Healthy Cities: Overview of a WHO International Program. Rev Environ Health (2000) 15(1-2):207–14. 10.1515/reveh.2000.15.1-2.207 10939093

[46] World Health Organization. National Healthy Cities Networks in the WHO European Region. Copenhagen: WHO Regional Office for Europe (2015).

[47] Denhardt J Terry L Ramirez-Delacruz E Andonoska L. Barriers to Citizen Engagement in Developing Countries. Int J Public Adm (2009) 32(14):1268–88. 10.1080/01900690903344726

[48] Valente M Crane A. Public Responsibility and Private Enterprise in Developing Countries. Calif Manage Rev (2010) 52(3):52–78. 10.1525/cmr.2010.52.3.52

[49] Jindra C Vaz A. Good Governance and Multidimensional Poverty: A Comparative Analysis of 71 Countries. Governance (2019) 32(4):657–75. 10.1111/gove.12394

[50] de Leeuw E. Intersectoral Action, Policy and Governance in European Healthy Cities. Public Health Panorama (2015) 1(2):111–204.

