# Supplementary material for: Urban Governance, Multisectoral Action, and Civic Engagement for Population Health, Wellbeing, and Equity in Urban Settings: A Systematic Review
Source: Int J Public Health. 2023 Aug 30;68:1605772. doi: 10.3389/ijph.2023.1605772 (PMC10500609; doi:10.3389/ijph.2023.1605772)
Supplement: Supplementary file 1 [file DataSheet1.docx]

**Supplementary material**

[I. Section S1. Search strategy 2](#_Toc98935924)

[II. Section S2. Supplementary results 6](#_Toc98935925)

[A. Table S1. Reasons for exclusion of studies after full–text review 7](#_Toc98935926)

[B. Excluded references 7](#_Toc98935927)

[C. Figure S1. A) Distribution of the target population among the included studies. B) Impact of participatory health governance in population health, equity, and well-being. C) Distribution of the reported indicators among the included studies 17](#_Toc98935929)

[E. Table S2. Indicators to assess the impact of participatory health governance on diverse policy domains 18](#_Toc98935930)

[F. Table S3. PRISMA Checklist 31](#_Toc98935931)

[III. Supplementary references 37](#_Toc98935932)

# Section S1. Search strategy

**TITLE**

**Urban governance, multisectoral action and civic engagement for population health, well-being, and equity in urban settings: a systematic review.**

- What are the validated and reliable indicators to assess intersectoral action and good governance for population health, wellbeing, and equity in LMIC urban settings?
- What are the civic engagement processes that facilitate good governance for population health, wellbeing, and equity in LMIC urban settings?

**Medline (Ovid)**

https://ovidsp.ovid.com/ovidweb.cgi?T=JS&NEWS=N&PAGE=main&SHAREDSEARCHID=4Z4WM6COxXSCAwl6O35WlUP5xTsqyvFmtTYgPQ3zF7z2o2bIigfwTHH9xhP4KzLe

Concepts 1-4 were combined using ‘AND’, limits 5) were combined using ‘NOT’

1. **Cities, urban settings, metropolitan areas**

(exp Cities/ or Urban Population/ or (urban setting* OR urban context* OR city OR cities OR metropolitan area* OR superblock* OR town OR towns OR municipal*).ab,ti,kf)

1. **Urban governance, health governance or civic engagement or multisectoral action**

(Health Policy/ or (((urban or health) adj3 govern*) or ((state* or health* or public or built environment*) adj3 polic*) or "health in all polic*" or "healthy public polic*").ab,ti,kf)

OR (Social Participation/ or Community Participation/ or Community Networks/ or (((community or public or citizen or civic or resident* or private or social or political) adj3 (particip* OR empower* OR involv* OR collaborat* OR engagement* OR implement* or partnership*)) or (participatory adj2 (approach* or framework*)) or participatory spaces or ((multisector* or multi-sector* or intersector* or inter-sector*) adj3 (action* or collaborat*)) or social citizenship or stakeholder*).ab,ti,kf)

1. **Health, well-being, equity**

(Urban Health/ or Health Equity/ or Healthcare Disparities/ or Health Status Disparities/ or "Social Determinants of Health"/ or Health Status Indicators/ or (wellbeing OR well-being OR health equit* OR urban health* OR quality of life OR community health* OR healthy cit* OR urban HEART OR health impact assessment* OR SDG11 OR "SDG 11" OR SDG3 OR "SDG 3" OR sustainable development goal* OR population health* OR residents health* OR healthy life OR healthy lives OR liveabil* OR livabil* OR ((city or cities or settlement*) AND (inclusive or safe or resilient or sustainab*))).ab,ti,kf)

1. **(Validated) theories, models, tools, instruments**

(indicator* OR determinant* OR tool OR tools OR instrument* OR factor OR factors OR intervention* OR definition* OR domain* OR model* OR theor* OR framework* OR concept* OR dimension* OR scor* OR index* OR indices OR scal* OR valid* OR value* OR evaluat* OR evidence OR assess* OR measure* OR metric* OR monitor* OR "Urban HEART").ab,ti,kf

1. **Limits: exclusion of animal studies**

not (exp animals/ not humans/)

**Embase.com**

1. **Cities, urban settings, metropolitan areas**

('city'/de or 'urban population'/de or ('urban setting*' OR 'urban context*' OR city OR cities OR 'metropolitan area*' OR superblock* OR town OR towns OR municipal*):ab,ti,kw)

1. **Urban governance, health governance or civic engagement or multisectoral action**

('health care policy'/de or (((urban or health) NEAR/3 govern*) or ((state* or health* or public or 'built environment*') NEAR/3 polic*) or 'health in all polic*' or 'healthy public polic*'):ab,ti,kw) OR ('social participation'/de or 'community participation'/de or 'community care'/de or (((community or public or citizen or civic or resident* or private or social or political) NEAR/3 (particip* OR empower* OR involv* OR collaborat* OR engagement* OR implement* or partnership*)) or (participatory NEAR/2 (approach* or framework*)) or 'participatory spaces' or ((multisector* or multi-sector* or intersector* or inter-sector*) NEAR/3 (action* or collaborat*)) or 'social citizenship' or stakeholder*):ab,ti,kw)

1. **Health, well-being, equity**

('urban health'/de or 'health equity'/de or 'health care disparity'/de or 'health disparity'/de or 'social determinants of health'/de or 'health status indicator'/de or (wellbeing OR well-being OR 'health equit*' OR 'urban health*' OR 'quality of life' OR 'community health*' OR 'healthy cit*' OR 'urban HEART' OR 'health impact assessment*' OR SDG11 OR 'SDG 11' OR SDG3 OR 'SDG 3' OR 'sustainable development goal*' OR 'population health*' OR 'residents health*' OR 'healthy life' OR 'healthy lives' OR liveabil* OR livabil* OR ((city or cities or settlement*) AND (inclusive or safe or resilient or sustainab*))):ab,ti,kw)

1. **(Validated) theories, models, tools, instruments**

(indicator* OR determinant* OR tool OR tools OR instrument* OR factor OR factors OR intervention* OR definition* OR domain* OR model* OR theor* OR framework* OR concept* OR dimension* OR scor* OR index* OR indices OR scal* OR valid* OR value* OR evaluat* OR evidence OR assess* OR measur* OR metric* OR monitor* OR 'Urban HEART'):ab,ti,kw

1. **Limits: exclusion of animal studies**

NOT ([animals]/lim NOT [humans]/lim) NOT ([Conference Abstract]/lim OR [Note]/lim)

**Cochrane Library**

1. **Cities, urban settings, metropolitan areas**

((urban NEXT setting* OR urban NEXT context* OR city OR cities OR metropolitan NEXT area* OR superblock* OR town OR towns OR municipal*):ab,ti,kw)

1. **Urban governance, health governance or civic engagement or multisectoral action**

((((urban or health) NEAR/3 govern*) or ((state* or health* or public or environment*) NEAR/3 polic*) or "health in all policy" OR "health in all policies" or "healthy public policy" OR "healthy public policies"):ab,ti,kw) OR ((((community or public or citizen or civic or resident* or private or social or political) NEAR/3 (particip* OR empower* OR involv* OR collaborat* OR engagement* OR implement* or partnership*)) or (participatory NEAR/2 (approach* or framework*)) or "participatory spaces" or ((multisector* or multi-sector* or intersector* or inter-sector*) NEAR/3 (action* or collaborat*)) or "social citizenship" or stakeholder*):ab,ti,kw)

1. **Health, well-being, equity**

((wellbeing OR well-being OR health NEXT equit* OR urban NEXT health* OR "quality of life" OR community NEXT health* OR healthy NEXT cit* OR "Urban HEART" OR health NEXT impact NEXT assess* OR SDG11 OR "SDG 11" OR SDG3 OR "SDG 3" OR "sustainable development goal" OR "sustainable development goals" OR population NEXT health* OR residents NEXT health* OR "healthy life" OR "healthy lives" OR liveabil* OR livabil* OR ((city or cities or settlement*) AND (inclusive or safe or resilient or sustainab*))):ab,ti,kw)

**Web of Science Core Collection**

1. **Cities, urban settings, metropolitan areas**

TS=(("urban setting*" OR "urban context*" OR city OR cities OR "metropolitan area*" OR superblock* OR town OR towns OR municipal*))

1. **Urban governance, health governance or civic engagement or multisectoral action**

TS=((((urban or health) NEAR/3 govern*) or ((state* or health* or public or built-environment*) NEAR/3 polic*) or "health in all polic*" or "healthy public polic*"))

OR TS=((((community or public or citizen or civic or resident* or private or social or political) NEAR/3 (particip* OR empower* OR involv* OR collaborat* OR engagement* OR implement* or partnership*)) or (participatory NEAR/2 (approach* or framework*)) or "participatory spaces" or ((multisector* or multi-sector* or intersector* or inter-sector*) NEAR/3 (action* or collaborat*)) or "social citizenship" or stakeholder*))

1. **Health, well-being, equity**

TS=((wellbeing OR well-being OR "health equit*" OR "urban health*" OR "quality of life" OR "community health*" OR "healthy cit*" OR "Urban HEART" OR "health impact assess*" OR SDG11 OR "SDG 11" OR SDG3 OR "SDG 3" OR "sustainable development goal*" OR "population health*" OR "residents health*" OR "healthy life" OR "healthy lives" OR liveabil* OR livabil* OR ((city or cities or settlement*) AND (inclusive or safe or resilient or sustainab*))))

1. **(Validated) theories, models, tools, instruments**

TS=(indicator* OR determinant* OR tool OR tools OR instrument* OR factor OR factors OR intervention* OR definition* OR domain* OR model* OR theor* OR framework* OR concept* OR dimension* OR scor* OR index* OR indices OR scal* OR valid* OR value* OR evaluat* OR evidence OR assess* OR measur* OR metric* OR monitor* OR "Urban HEART")

1. **Limits: Document types, Web of Science Categories**

Refined by: DOCUMENT TYPES: (ARTICLE OR EARLY ACCESS OR EDITORIAL MATERIAL OR LETTER OR REVIEW OR BOOK CHAPTER) AND WEB OF SCIENCE CATEGORIES: (POLITICAL SCIENCE OR PUBLIC ENVIRONMENTAL OCCUPATIONAL HEALTH OR URBAN STUDIES OR HEALTH POLICY SERVICES OR MEDICINE GENERAL INTERNAL OR HEALTH CARE SCIENCES SERVICES OR SOCIAL SCIENCES INTERDISCIPLINARY)

**Google scholar** (First 200 results according to relevance ranking, out of a total of 11’700 results)

1. **Focus: Determinants and Indicators of urban health**

"urban setting|settings|context|governance"|city|cities|superblocks|metropolitan "urban|population health"|"health equity"|"healthy city|cities"|"urban HEART"|SDG11|"SDG 11"|SDG3|"SDG 3"|sustainable|healthy|inclusive|safe|resilient indicators|determinants

**Global Health (Ovid)**

**1) Cities, urban settings, metropolitan areas**

(exp Cities/ or Towns/ or Urban Population/ or (urban setting* OR urban context* OR city OR cities OR megacities OR metropolitan area* OR superblock* OR town OR towns OR municipal*).ab,ti,id)

**2) Urban governance, health governance or civic engagement or multisectoral action**

(Health Policy/ or (((urban or health) adj3 govern*) or ((state* or health* or public or built environment*) adj3 polic*) or "health in all polic*" or "healthy public polic*").ab,ti,id)

OR

(Social Participation/ or Community Involvement/ or Community Action/ or **(((community or public or citizen or civic or resident* or private or social or political) adj3 (particip* OR empower* OR involv* OR collaborat* OR engagement* OR implement* or partnership*)) or (**participatory adj2 (approach* or framework*)) or participatory spaces or ((multisector* or multi-sector* or intersector* or inter-sector*) adj3 (action* or collaborat*)) or social citizenship or stakeholder*).ab,ti,id)

**3) Health, Wellbeing, Equity**

(Community Health/ or Health Inequalities/ or (wellbeing OR well-being OR health equit* OR urban health* OR quality of life OR community health* OR healthy cit* OR "urban HEART" OR health impact assessment* OR SDG11 OR "SDG 11" OR SDG3 OR "SDG 3" OR sustainable development goal* OR population health* OR residents health* OR healthy life OR healthy lives OR liveabil* OR livabil* OR ((city or cities or settlement*) AND (inclusive or safe or resilient or sustainab*))).ab,ti,id)

**4) (validated) theories, models, tools, instruments**

(indicator* OR determinant* OR tool OR tools OR instrument* OR factor OR factors OR intervention* OR definition* OR domain* OR model* OR theor* OR framework* OR concept* OR dimension* OR scor* OR index* OR indices OR scal* OR valid* OR value* OR evaluat* OR evidence OR assess* OR measure* OR metric* OR monitor* OR "Urban HEART").ab,ti,id

# Section S2. Supplementary results

## **Table S1. Reasons for exclusion of studies after full–text review**

| Exclusion criteria | Number of excluded studies | References |
| --- | --- | --- |
| The study did not assess participatory health governance | 42 | 1, 5, 7, 8, 21- 24, 26-28, 30-35, 38, 40, 41, 46, 52, 60, 62, 68, 70-72, 81, 85, 87, 90, 92, 95, 97-103,105 |
| The study did not use a standardized tool to assess participatory health governance | 68 | 1, 2, 4-6, 9-12, 14-16, 19-24, 26-34, 36, 38-41, 43, 44, 46, 48, 51, 53, 55, 56, 59-61, 65, 67, 70, 71, 74, 75, 77, 80, 81, 83, 85, 87, 89-95, 97, 99, 102- 105 |
| The study did not follow a standardized process to identify/create indicators | 53 | 1, 2, 5, 6, 9, 11, 12, 14-16, 19-24, 26-31, 33, 34, 36, 38-41, 43, 44, 46, 53, 56, 59, 68, 70, 71, 75, 80, 81, 83, 87, 89-95, 99, 102, 104 |
| The outcomes are not population health, wellbeing and/or equity | 46 | 3, 5, 12, 20, 22-24, 31-33, 44, 49, 50, 52, 54, 58, 65-71, 73, 74, 77, 79-81, 83-85, 87-92, 94-97, 99-101, 105 |
| The aim of the study was not the creation of a set of indicators | 38 | 3, 5, 12, 20, 22-24, 31, 33, 52, 54, 58, 67-71, 73,74, 77, 79-83, 88-91, 94-97, 99-101, 105 |
| The does not present individual results from at least one city | 44 | 4, 5, 10, 13, 15, 17, 18, 25, 36, 37, 41, 42, 45-49, 54, 57, 59, 61-64, 68-70, 71, 74-79, 81, 82, 86-92, 104 |

## **Excluded references**

1. Ahari SS, Habibzadeh S, Yousefi M, Amani F, Abdi R. Community based needs assessment in an urban area: a participatory action research project. BMC Public Health 2012; 12: 161.

2. Arteaga E, Rice M, Franceschini MC. Documenting the current state of the Healthy Municipalities, Cities and Communities initiative in the Americas. International Quarterly of Community Health Education 2007; 28(4): 277-88.

3. Bartoletti R, Faccioli F. Civic Collaboration and Urban Commons. Citizen's voices on a Public Engagement Experience in an Italian City. Partecip Confl 2020; 13(2): 1132-+.

4. Batista LE, Barros S, Silva NG, Tomazelli PC, da Silva A, Rinehart D. Indicators for monitoring and evaluating the implementation of the National Policy for the Integrative Health of the Black Population. Saude Soc 2020; 29(3): 18.

5. Baum F, Jolley G, Hicks R, Saint K, Parker S. What makes for sustainable Healthy Cities initiatives?--A review of the evidence from Noarlunga, Australia after 18 years. Health Promotion International 2006; 21(4): 259-65.

6. Becker D, Edmundo KB, Guimaraes W, et al. Network of healthy communities of Rio de Janeiro--Brazil. Promot Educ 2007; 14(2): 101-2.

7. Begun JW, Kahn LM, Cunningham BA, Malcolm JK, Potthoff S. A Measure of the Potential Impact of Hospital Community Health Activities on Population Health and Equity. J Public Health Manag Pract 2018; 24(5): 417-23.

8. Bhatia R, Corburn J. Lessons from San Francisco: health impact assessments have advanced political conditions for improving population health. Health Aff (Millwood) 2011; 30(12): 2410-8.

9. Binet A, Gavin V, Carroll L, Arcaya M. Designing and Facilitating Collaborative Research Design and Data Analysis Workshops: Lessons Learned in the Healthy Neighborhoods Study. Int J Environ Res Public Health 2019; 16(3): 24.

10. Browne AJ, Varcoe CM, Wong ST, et al. Closing the health equity gap: evidence-based strategies for primary health care organizations. Intern 2012; 11: 59.

11. Browne GR, Davern MT, Giles-Corti B. An analysis of local government health policy against state priorities and a social determinants framework. Australian and New Zealand journal of public health 2016; 40(2): 126-31.

12. Burton S. Evaluation of healthy city projects: stakeholder analysis of two projects in Bangladesh. Environ Urban 1999; 11(1): 41-52.

13. Donchin M, Shemesh AA, Horowitz P, Daoud N. Implementation of the Healthy Cities' principles and strategies: an evaluation of the Israel Healthy Cities network. Health Promotion International 2006; 21(4): 266-73.

14. Fehr R, Fertmann R, Stender KP, Lettau N, Trojan A. Urban Health (StadtGesundheit): The Wider Perspective Exemplified by the City State of Hamburg. Gesundheitswesen 2016; 78(8-9): 498-504.

15. Flynn BC, Ray DW, Rider MS. Empowering communities: action research through healthy cities. Health education quarterly 1994; 21(3): 395-405.

16. Flynn BC, Rider M, Ray DW. Healthy cities: the Indiana model of community development in public health. Health education quarterly 1991; 18(3): 331-47.

17. Fortune K, Becerra-Posada F, Buss P, et al. Health promotion and the agenda for sustainable development, WHO Region of the Americas. Bull World Health Organ 2018; 96(9): 621-6.

18. Fosse E, Sherriff N, Helgesen M. Leveling the Social Gradient in Health at the Local Level: Applying the Gradient Equity Lens to Norwegian Local Public Health Policy. International journal of health services : planning, administration, evaluation 2019; 49(3): 538-54.

19. Freire M, Sa R, Gurgel IGD. Healthier Saire: a intersectorial policy as a turning point for local equity. Cienc 2017; 22(12): 3893-902.

20. Froding K, Geidne J, Elander I, Eriksson C. Towards sustainable structures for neighbourhood development? Healthy city research in four Swedish municipalities 2003-2009. J Health Organ Manag 2013; 27(2): 225-45.

21. Fryer P. A healthy city strategy three years on - The case of Oxford City Council. Health Promotion 1988; 3(2): 213-8.

22. Gamache S, Diallo TA, Shankardass K, Lebel A. The Elaboration of an Intersectoral Partnership to Perform Health Impact Assessment in Urban Planning: The Experience of Quebec City (Canada). Int J Environ Res Public Health 2020; 17(20): 17.

23. Gerez Valls MD, Velázquez Valoria I. The health of cities and their citizens (urban development and municipal public health). 2008 SESPAS Report. Gaceta Sanitaria 2008; 22(SUPPL. 1): 71-8.

24. Gjorgjev D, Dimovska M, Morris G, Howie J, Popovska MB, Latkovikj MT. How good is our place—implementation of the place standard tool in North Macedonia. International Journal of Environmental Research and Public Health 2020; 17(1).

25. Glismann W, Trojan A, Süß W. Integrated sustainability - Oriented reporting - Key indicators for counties and cities. Gesundheitswesen 2005; 67(2): 150-4.

26. Gorman D, Douglas MJ, Conway L, Noble P, Hanlon P. Transport policy and health inequalities: a health impact assessment of Edinburgh's transport policy. Public Health 2003; 117(1): 15-24.

27. Grant M. European Healthy City Network Phase V: patterns emerging for healthy urban planning. Health promotion international 2015; 30: i54-i70.

28. Green G, Acres J, Price C, Tsouros A. City health development planning. Health promotion international 2009; 24 Suppl 1: i72-i80.

29. Harpham T, Burton S, Blue I. Healthy city projects in developing countries: the first evaluation. Health Promotion International 2001; 16(2): 111-25.

30. Harpham T, Few R. The Dar Es Salaam Urban Health Project, Tanzania: a multi-dimensional evaluation. J Public Health Med 2002; 24(2): 112-9.

31. Higgs C, Badland H, Simons K, Knibbs LD, Giles-Corti B. The Urban Liveability Index: developing a policy-relevant urban liveability composite measure and evaluating associations with transport mode choice. International journal of health geographics 2019; 18(1): 14.

32. Ho HC, Wong MS, Man HY, Shi Y, Abbas S. Neighborhood-based subjective environmental vulnerability index for community health assessment: Development, validation and evaluation. Sci Total Environ 2019; 654: 1082-90.

33. Holscher K, Frantzeskaki N, McPhearson T, Loorbach D. Tales of transforming cities: Transformative climate governance capacities in New York City, U.S. and Rotterdam, Netherlands. J environ manage 2019; 231: 843-57.

34. Hu SC, Kuo HW. The development and achievement of a healthy cities network in Taiwan: sharing leadership and partnership building. Glob Health Promot 2016; 23(1 Suppl): 8-17.

35. Ison E. Health impact assessment in a network of European cities. Journal of urban health : bulletin of the New York Academy of Medicine 2013; 90: 105-15.

36. Jabot F, Tremblay E, Rivadeneyra A, Diallo TA, Lapointe G. A Comparative Analysis of Health Impact Assessment Implementation Models in the Regions of Monteregie (Quebec, Canada) and Nouvelle-Aquitaine (France). Int J Environ Res Public Health 2020; 17(18): 09.

37. Jackisch J, Zamaro G, Green G, Huber M. Is a healthy city also an age-friendly city? Health promotion international 2015; 30: i108-i17.

38. James P, Ito K, Buonocore JJ, Levy JI, Arcaya MC. A health impact assessment of proposed public transportation service cuts and fare increases in Boston, Massachusetts (U.S.A.). Int J Environ Res Public Health 2014; 11(8): 8010-24.

39. Janzen C, Marko J, Schwandt M. Embedding health equity strategically within built environments. Can J Public Health-Rev Can Sante Publ 2018; 109(4): 590-7.

40. Johnson Thornton RL, Greiner A, Fichtenberg CM, Feingold BJ, Ellen JM, Jennings JM. Achieving a healthy zoning policy in Baltimore: results of a health impact assessment of the TransForm Baltimore zoning code rewrite. Public Health Rep 2013; 128 Suppl 3: 87-103.

41. Johnstone PW. A case study of new approaches to address health inequalities: Due North five years on. Br Med Bull 2019; 132(1): 17-31.

42. Kang E. Intersectoral collaboration for physical activity in Korean Healthy Cities. Health Promotion International 2016; 31(3): 551-61.

43. Kang E, Park HJ, Kim JE. Health impact assessment as a strategy for intersectoral collaboration. J Prev Med Pub Health 2011; 44(5): 201-9.

44. Karimi J, Holakouie Naieni K, Ahmadnezhad E. Community assessment of Shahin-Shar, Isfahan, I. R. Iran to develop community health action plan. Iranian Journal of Epidemiology 2012; 8(1): 21-30.

45. Kasmel A, Andersen PT. Measurement of community empowerment in three community programs in Rapla (Estonia). Int J Environ Res Public Health 2011; 8(3): 799-817.

46. Kegler MC, Twiss JM, Look V. Assessing community change at multiple levels: the genesis of an evaluation framework for the California Healthy Cities Project. Health education & behavior : the official publication of the Society for Public Health Education 2000; 27(6): 760-79.

47. Kelly C, George J, Lanman ER. Colorado Healthy Eating and Active Living Cities and Towns Campaign. Am J Prev Med 2018; 54(5 Suppl 2): S145-S9.

48. Kim S, Flaskerud JH, Koniak-Griffin D, Dixon EL. Using community-partnered participatory research to address health disparities in a Latino community. J Prof Nurs 2005; 21(4): 199-209.

49. Kim Y, Lee SJ. The Development and Application of a Community Wellbeing Index in Korean Metropolitan Cities. Soc Indic Res 2014; 119(2): 533-58.

50. Kumaresan J, Prasad A, Alwan A, Ishikawa N. Promoting health equity in cities through evidence-based action. Journal of urban health : bulletin of the New York Academy of Medicine 2010; 87(5): 727-32.

51. Kusworo, Rochmansjah H. The implementation of no-smoking area policy to improve community health and its relationship to the Country’s pharmaceutical policy: A study in Semarang City in Indonesia. Journal of Global Pharma Technology 2020; 12(4): 31-40.

52. Lee CB, Huang NC, Kung SF, Hu SC. Opportunity for HiAP through a Healthy Cities initiative in Taiwan: a multiple streams analysis. Health promotion international 2021; 36(1): 78-88.

53. Lillefjell M, Magnus E, Knudtsen MS, et al. Governance for public health and health equity: The Trondelag model for public health work. Scand J Public Health 2018; 46(22_suppl): 37-47.

54. Lipp A, Winters T, de Leeuw E. Evaluation of partnership working in cities in phase IV of the WHO Healthy Cities Network. Journal of urban health : bulletin of the New York Academy of Medicine 2013; 90: 37-51.

55. Lowe M, Arundel J, Hooper P, et al. Liveability aspirations and realities: Implementation of urban policies designed to create healthy cities in Australia. Soc Sci Med 2020; 245: 112713.

56. Lowe M, Whitzman C, Badland H, et al. Planning Healthy, Liveable and Sustainable Cities: How Can Indicators Inform Policy? Urban Policy Res 2015; 33(2): 131-44.

57. McClellan CS. Utilizing a national performance standards local public health assessment instrument in a community assessment process: the Clarendon County Turning Point Initiative. Journal of public health management and practice : JPHMP 2005; 11(5): 428-32.

58. Mehdipanah R, Schulz AJ, Israel BA, et al. Urban HEART Detroit: a Tool To Better Understand and Address Health Equity Gaps in the City. Journal of urban health : bulletin of the New York Academy of Medicine 2018; 95(5): 662-71.

59. Mendes R, Falvo F. Motuca healthy municipality project: building together a better future. Promotion & education 2007; 14(2): 81-2.

60. Metzler MM, Higgins DL, Beeker CG, et al. Addressing urban health in Detroit, New York City, and Seattle through community-based participatory research partnerships. Am J Public Health 2003; 93(5): 803-11.

61. Miro A, Kishchuk NA, Perrotta K, Swinkels HM. Healthy Canada by Design CLASP: Lessons learned from the first phase of an intersectoral, cross-provincial, built environment initiative. Can J Public Health 2014; 106(1 Suppl 1): eS50-63.

62. Moon JY, Nam EW, Dhakal S. Empowerment for Healthy Cities and communities in Korea. Journal of urban health : bulletin of the New York Academy of Medicine 2014; 91(5): 886-93.

63. Nunez A, Colomer C, Peiro R, Alvarez-Dardet C. The Valencian Community Healthy Cities network: Assessment of the implementation process. Health Promotion International 1994; 9(3): 189-98.

64. Ouellet F, Durand D, Forget G. Preliminary results of an evaluation of three healthy cities initiatives in the Montreal area. Health Promotion International 1994; 9(3): 153-9.

65. Pagliccia N, Spiegel J, Alegret M, Bonet M, Martinez B, Yassi A. Network analysis as a tool to assess the intersectoral management of health determinants at the local level: a report from an exploratory study of two Cuban municipalities. Soc Sci Med 2010; 71(2): 394-9.

66. Patterson L, Heller R, Robinson J, et al. Developing a European urban health indicator system: results of EURO-URHIS 1. Eur J Public Health 2017; 27(suppl_2): 4-8.

67. Plochg T, Schmidt M, Klazinga NS, Stronks K. Health governance by collaboration: a case study on an area-based programme to tackle health inequalities in the Dutch city of the Hague. Eur J Public Health 2013; 23(6): 939-46.

68. Plumer KD, Kennedy L, Trojan A. Evaluating the implementation of the WHO Healthy Cities Programme across Germany (1999-2002). Health Promotion International 2010; 25(3): 342-54.

69. Plumer KD, Trojan A. ["Healthy cities"--requirements and performance. Questionnaire results and a suggestion on quality monitoring (healthy-cities-barometer)]. Gesundheitswesen 2004; 66(3): 202-7.

70. Prasad A. The Urban Health Equity Assessment and Response Tool (HEART)—a decade of development and implementation. J Urban Health 2018.

71. Prasad A, Gray CB, Ross A. Metrics in urban health: current developments and future prospects. Annual review of public … 2016.

72. Prasad A, Groot AM, Monteiro T, et al. Linking evidence to action on social determinants of health using Urban HEART in the Americas. Rev Panam Salud Publica 2013; 34(6): 407-15.

73. Prasad A, Kano M, Dagg KA, et al. Prioritizing action on health inequities in cities: An evaluation of Urban Health Equity Assessment and Response Tool (Urban HEART) in 15 cities from Asia and Africa. Soc Sci Med 2015; 145: 237-42.

74. Ramirez-Rubio O, Daher C, Fanjul G, et al. Urban health: an example of a "health in all policies" approach in the context of SDGs implementation. Global health 2019; 15(1): 87.

75. Reddy S, Paode P, Speer M, Semenchuk N, Goble K, White A. Moving the needle towards health equity: A policy-driven transdisciplinary approach to address health disparities for vulnerable communities. Health Education Journal 2018; 77(8): 1018-24.

76. Reid A, Abraczinskas M, Scott V, et al. Using Collaborative Coalition Processes to Advance Community Health, Well-Being, and Equity: A Multiple-Case Study Analysis From a National Community Transformation Initiative. Health Educ Behav 2019; 46(1_suppl): 100S-9S.

77. Ritsatakis A. Equity and social determinants of health at a city level. Health promotion international 2009; 24 Suppl 1: i81-i90.

78. Ritsatakis A. Equity and the social determinants of health in European cities. Journal of urban health : bulletin of the New York Academy of Medicine 2013; 90: 92-104.

79. Ritsatakis A, Ostergren PO, Webster P. Tackling the social determinants of inequalities in health during Phase V of the Healthy Cities Project in Europe. Health promotion international 2015; 30: i45-i53.

80. Schmidt M, Joosen I, Kunst AE, Klazinga NS, Stronks K. Generating political priority to tackle health disparities: a case study in the Dutch city of The Hague. Am J Public Health 2010; 100 Suppl 1: S210-5.

81. Shadpour K. The Healthy Cities Project in the Islamic Republic of Iran. Urban Health Newsl 1996; (28): 20-5.

82. Simos J, Spanswick L, Palmer N, Christie D. The role of health impact assessment in Phase V of the Healthy Cities European Network. Health Promotion International 2015; 30 Suppl 1: i71-i85.

83. Sperandio AM, Francisco LLF, Mattos TP. [Health promotion policy and urban planning: joint efforts for the development of healthy cities]. Cienc 2016; 21(6): 1931-8.

84. Storm I, Harting J, Stronks K, Schuit AJ. Measuring stages of health in all policies on a local level: the applicability of a maturity model. Health Policy 2014; 114(2-3): 183-91.

85. Takano T, Nakamura K. An analysis of health levels and various indicators of urban environments for Healthy Cities projects. J Epidemiol Community Health 2001; 55(4): 263-70.

86. Tallarek nee Grimm MJ, Helgesen MK, Fosse E. Reducing social inequities in health in Norway: concerted action at state and local levels? Health Policy 2013; 113(3): 228-35.

87. Torres M, Silva LT, Santos L, Mendes JFG. Health and well-being in urban environment: From policies to pratice. Revista Portuguesa de Saude Publica 2013; 31(1): 95-107.

88. Tsouros A. City leadership for health and sustainable development: the World Health Organization European Healthy Cities Network. Health Promotion International 2009; 24 Suppl 1: i4-i10.

89. Tsouros A. City leadership for health and well-being: back to the future. J Urban Health 2013; 90 Suppl 1: 4-13.

90. Tsouros A, de Leeuw E, Green G. Evaluation of the Fifth Phase (2009-2013) of the WHO European Healthy Cities Network: further sophistication and challenges. Health Promotion International 2015; 30 Suppl 1: i1-i2.

91. Van Vliet J. How to apply the evidence-based recommendations for greater health equity into policymaking and action at the local level? Scand J Public Health 2018; 46(22): 28-36.

92. Webster P, Sanderson D. Healthy Cities indicators--a suitable instrument to measure health? J Urban Health 2013; 90 Suppl 1: 52-61.

93. Weech-Maldonado R, Merrill SB. Building partnerships with the community: lessons from the Camden Health Improvement Learning Collaborative. J Healthc Manag 2000; 45(3): 189-205.

94. Wehrens R, Bekker M, Bal R. The construction of evidence-based local health policy through partnerships: Research infrastructure, process, and context in the Rotterdam 'Healthy in the City' programme. J Public Health Policy 2010; 31(4): 447-60.

95. Yang J, Luo X, Xiao Y, Shen S, Su M, Bai Y. Comparing the Use of Spatially Explicit Indicators and Conventional Indicators in the Evaluation of Healthy Cities: A Case Study in Shenzhen, China. International journal of … 2020.

96. Yassi A, Fernandez N, Fernandez A, Bonet M, Tate RB, Spiegel J. Community participation in a multisectoral intervention to address health determinants in an inner-city community in central Havana. J Urban Health 2003; 80(1): 61-80.

97. Babajide O, Martins DC, Maani N, Abdalla SM, Gomez EJ, Pongsiri MJ, et al. Improving Decision-Making for Population Health in Nonhealth Sectors in Urban Environments: the Example of the Transportation Sector in Three Megacities-the 3-D Commission. J Urban Health. 2021;98(Suppl 1):60-8.

98. Lee A, Nakamura K. Engaging Diverse Community Groups to Promote Population Health through Healthy City Approach: Analysis of Successful Cases in Western Pacific Region. Int J Environ Res Public Health. 2021;18(12):19.

99. Lowe M, Adlakha D, Sallis JF, Salvo D, Cerin E, Moudon AV, et al. City planning policies to support health and sustainability: an international comparison of policy indicators for 25 cities. Lancet Glob Health. 2022;10(6):e882-e94.

100. Patterson L, Heller R, Robinson J, Birt CA, Ameijden Ev, Bocsan I, et al. Developing a European urban health indicator system: results of EURO-URHIS 1. (Special Issue: Influencing policy to maximise health gain in European cities.). European Journal of Public Health. 2017;27(Suppl. 2):4-8.

101. Pope D, Puzzolo E, Birt CA, Guha J, Higgerson J, Patterson L, et al. Collecting standardised urban health indicator data at an individual level for adults living in urban areas: methodology from EURO-URHIS 2. (Special Issue: Influencing policy to maximise health gain in European cities.). European Journal of Public Health. 2017;27(Suppl. 2):42-9.

102. Shafique S, Bhattacharyya DS, Hossain M, Hasan SM, Ahmed S, Islam R, et al. Strengthening health service delivery and governance through institutionalizing ‘Urban Health Atlas’ - a geo-referenced Information Communication and Technology tool: lessons learned from an implementation research in three cities in Bangladesh. 2022.

103. Stauber C, Weaver SR, Dai D, Luo R, Rothenberg R. Comparison of cities using the urban health index: an analysis of demographic and health survey data from 2003-2013. Comparison of cities using the urban health index: an analysis of demographic and health survey data from. 2003;75.

104. Watson-Thompson J, May MJ, Jefferson J, Young Y, Young A, Schultz J. Examining the contributions of a community coalition in addressing urban health determinants. (Special Issue: Exemplars of community-based research on health disparities.). Journal of Prevention and Intervention in the Community. 2018;46(1):7-27.

105. Yan CT, McClay CJ, Maharaj M, Gray H, Muhammad N, Lewis SM, et al. "Don't shoot, I want to grow up": findings of a multicity youth-led health assessment. Progress in Community Health Partnerships: Research, Education and Action. 2021;15(3):297-318.

## **Figure S1. A) Distribution of the target population among the included studies. B) Impact of participatory health governance in population health, equity, and well-being. C) Distribution of the reported indicators among the included studies**

**
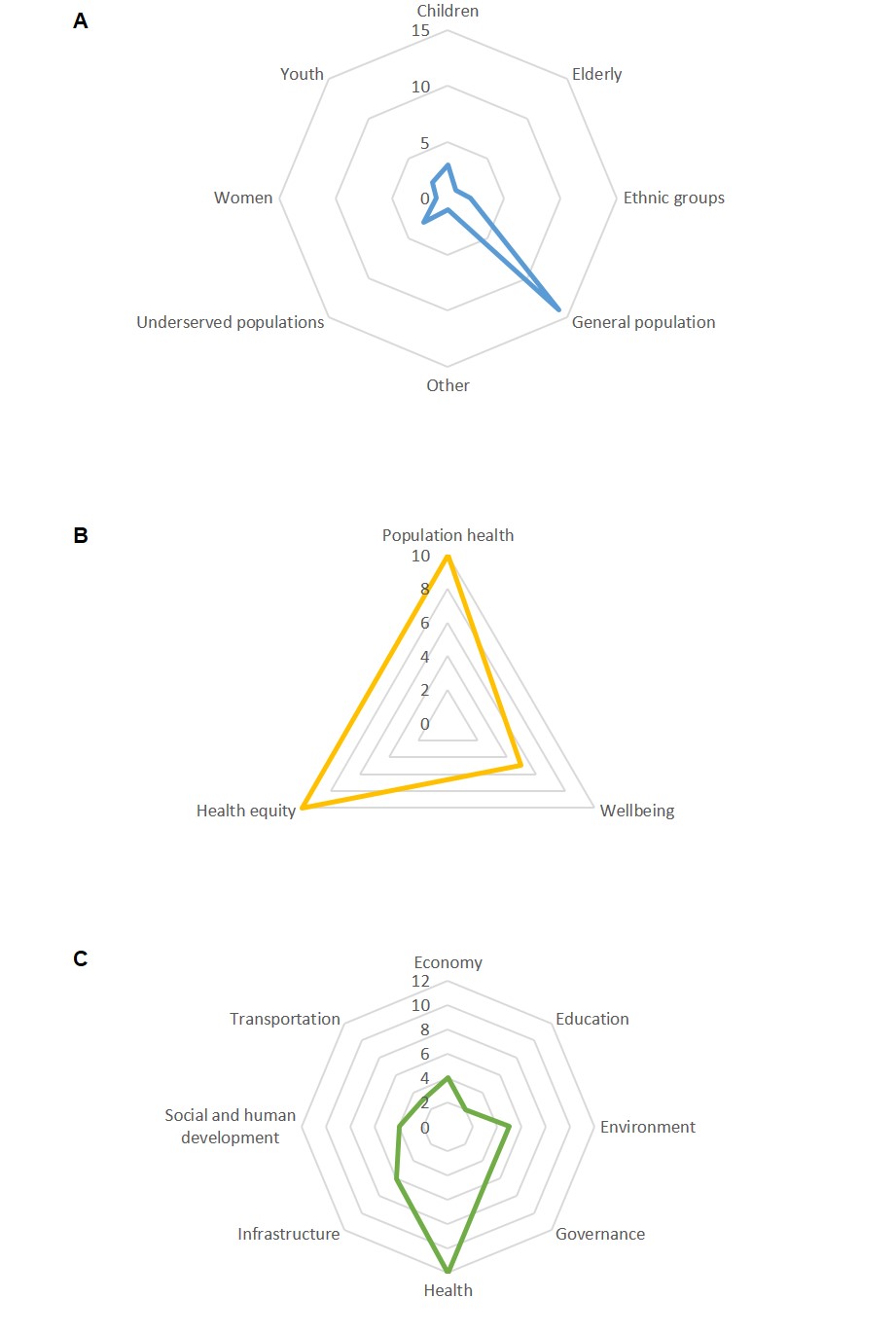
**

## **Table S2. Indicators to assess the impact of participatory health governance on diverse policy domains**

| Indicators | Definition |
| --- | --- |
| HEALTH/HEALTHCARE | |
| Improvement in parental skills (%) *(F. Baum, 1992)* (1) | Percentage of participants improving their parental skills. |
| Reduction in children's negative behaviours (%) *(F. Baum, 1992)* (1) | Percentage of children with less negative behaviours. |
| Stress reduction (%) *(F. Baum, 1992)* (1) | Percentage of parents with reduced stress. |
| Increase of social support (%) *(F. Baum, 1992)* (1) | Percentage of parents receiving social support. |
| Medical doctors in primary health care (number per 1000 population) *(A. Freitas, 2020)* (2) | Medical doctors in primary health care (number per 1000 population). |
| Nurses in primary health care (number per 1000 population) *(A. Freitas, 2020)* (2) | Nurses in primary health care (number per 1000 population). |
| Maternal consultations (number per 1000 live births) *(A. Freitas, 2020)* (2) | Maternal consultations (number per 1000 live births). |
| Infant mortality rate (number per 1000 births) *(L. O. Andrade, 2005; V. Junqueira, 2002; K. Makadzange, 2018)* (3–5) | Probability of dying between birth and age 1 per 1000 live births. |
| Neo-natal mortality *(V. Junqueira, 2002)* (4) | Number of deaths during the first 28 completed days of life per 1000 live births in a given year or another period. |
| Post-natal mortality rate (per 1000 living births) *(V. Junqueira, 2002)* (4) | Number of resident newborns dying between 28 and 364 days of age in a specified geographic area. |
| Under five mortality rate (number per 1000 births) *(K. Makadzange, 2018)* (5) | Probability of dying by age 5 per 1000 live births. |
| Maternal mortality ratio (per 100,000 live births) *(K. Makadzange, 2018)* (5) | The maternal mortality ratio is defined as the number of maternal deaths during a given period per 100,000 live births during the same period. |
| Fully immunized children (%) *(K. Makadzange, 2018)* (5) | The percentage of one-year-olds who have received one dose of Bacille Calmette-Guérin (BCG) vaccine, three doses of polio vaccine, three doses of the combined diphtheria, tetanus toxoid and pertussis (DTP3) vaccine, and one dose of measles vaccine. |
| Skilled birth attendance (%) *(K. Makadzange, 2018)* (5) | The proportion of births attended by skilled health personnel. |
| Contraception use 15 to 49 years (%) *(K. Makadzange, 2018; F. Baum, 1992)* (1,5) | Percentage of women aged 15 to 49 years who use contraception. |
| Contraception use 15 to 39 years (%) *(F. Baum, 1992)* (1) | Percentage of men aged 15 to 39 years who use contraception. |
| Good mental health (% of residents) *(R. Mehdipanah, 2021)* (6) | Percentage of adults (aged 18 and over) reporting good mental health was derived by subtracting crude prevalence rates of individuals with poor mental health from 100. |
| No asthma (% of residents) *(R. Mehdipanah, 2021)* (6) | Percentage of adults (aged 18 and over) without asthma was derived by subtracting crude prevalence rates of individuals with asthma from 100. |
| Not disabled (% of residents) *(R. Mehdipanah, 2021)* (6) | Percentage of adults (aged 18 and over) without disability was derived by subtracting the total individuals aged 18–64 who did not report any difficulties with vision, hearing, ambulatory, cognitive, self-care, and independent living from the total population in the same age group. |
| Older adults reporting limitations/disabilities (%) *(A. Freitas, 2020)* (2) | Older adults reporting limitations/disabilities (%). |
| Healthcare status *(R. Mehdipanah, 2021)* (6) | Percentage with health insurance was derived by dividing the total number of adults with public or private insurance by the total adult population. |
| Premature mortality rate per 10,000 inhabitants *(A. M. Novoa, 2018)* (7) | Premature mortality rate per 10,000 inhabitants. |
| Tuberculosis rate per 100,000 inhabitants *(A. M. Novoa, 2018)* (7) | Tuberculosis rate expressed as number per 100,000 inhabitants. |
| Gonococcal rate per 100,000 inhabitants *(A. M. Novoa, 2018)* (7) | Gonococcal rate expressed as number per 100,000 inhabitants. |
| Teenage fecundity rate per 1000 girls aged 15 to 19 years old *(A. M. Novoa, 2018) ** (7) | Teenage fecundity rate expressed as number per 1000 girls aged 15 to 19 years old. |
| Problematic drug consumption index *(A. M. Novoa, 2018)* (7) | Compound index that includes 4 indicators: rate of new treatments due to drug consumption, mortality rate due to drug overdose, rate of emergency visits among drug consumers, number of syringes found on the street. |
| Self-rate health in young people (14-25 years old) from disadvantaged neighbourhoods after occupational training (%) *(F. Baum, 1992)* (1) | Percentage of young people experimenting improvements in self-rate health after occupational training. |
| Self-esteem in young people (14-25 years old) from disadvantaged neighbourhoods after occupational training (%) *(F. Baum, 1992)* (1) | Percentage of young people experimenting improvements in self-esteem after occupational training. |
| Improvement in mental health, emotional well-being and perceived social support in participants aged 25 to 65 years after participation in free workshops (%) *(F. Baum, 1992)* (1) | Percentage of people aged 25 to 65 who have had improvements in mental health, emotional well-being, and perceived social support after participating in one or more free workshops. |
| Improvement in mental health, social inclusion, and empowerment among immigrant adolescent girls (12-16 years old) due to sport participation and group dynamics (%) *(F. Baum, 1992)* (1) | Percentage of immigrant adolescent girls whose mental health and social inclusion improved, and who have been empowered due to their participation in sports and group dynamics. |
| Improvement in perceived health of elder people (≥ 59 years) living in isolation (%) *(F. Baum, 1992)* (1) | Percentage of older adults who experienced improvements in perceived health while living in isolation. |
| Improvement in mental health of elder people (≥ 59 years) living in isolation (%) *(F. Baum, 1992)* (1) | Percentage of older adults who experienced improvements in mental health while living in isolation. |
| Reduction of psychological distress of elder people (≥ 59 years) living in isolation (%) *(F. Baum, 1992)* (1) | Percentage of older adults who experimented a reduction in psychological distress while living in isolation. |
| Older adults living alone and in social isolation *(A. Freitas, 2020)* (2) | Number of older adults living alone and in social isolation. |
| Eating unhealthy foods to deal with stress (never/rarely/occasionally/most days/every day) *(C.Riley, 2021)* (8) | Frequency in which they eat unhealthy foods to deal with stress. |
| Having difficulty sleeping (never/rarely/occasionally/most days/every day) *(C.Riley, 2021)* (8) | Frequency in which people have difficulty sleeping. |
| Smoking or drinking alcohol to deal with stress (never/rarely/occasionally/most days/every day) *(C.Riley, 2021)* (8) | Frequency in which people either smoke or drink alcohol to deal with stress. |
|  | |
| TRANSPORTATION | |
| Pedestrian accidents (number) *(A. Freitas, 2020)* (2) | Number of pedestrian accidents. |
| Fatality rate due to road traffic accidents (number per 100,000 inhabitants; number per 1000 victims) *(L. O. Andrade, 2005; A. Freitas, 2020)* (2,3) | Estimated road traffic fatal injury deaths per 1000 population. Relation between the number of deaths in a travel accident and the total number of victims (dead/injured) in travel accidents. |
| Non-auto commuters *(R. Mehdipanah, 2021)* (6) | Percentage of non-auto commuters was derived by dividing the total number of non-auto commuters (walked, biked, or used public transportation) by the total population who commute for work to obtain the percentage. |
| Vehicle km travelled *(A. M. Novoa, 2018)* (7) | Kilometres travelled in a vehicle. |
| Sustainable and safe transportation *(L. Farhang, 2008)* (9) | Transport that can be sustained given certain limitations in time and space set by the environment and/or by certain demands of society. |
| Walkability index *(A. Freitas, 2020)* (2) | The Walkability Index is intended to help address a growing demand for data products and tools that enable users to consistently compare multiple places based on their suitability for walking as a means of travel. It may be of use as source data for transportation or land use sketch planning tools. |
| Population using public transportation and soft modes of mobility (%) (A. Freitas, 2020) (2) | Percentage of population using public transportation and soft modes of mobility. |
| Average commute time to work or study (minutes) *(A. Freitas, 2020)* (2) | Average commute time to work or study (minutes). |
| Ease of moving about by biking or walking (very easy/moderately ease/moderately difficult, difficult) *(C.Riley, 2021)* (8) | Proportion of people that consider moving about by biking or walking very easy, moderately easy, moderately difficult or difficult. |
| Ease of moving about by public transportation (very easy/moderately ease/moderately difficult, difficult) *(C.Riley, 2021)* (8) | Proportion of people that consider moving about by p very easy, moderately easy, moderately difficult or difficult. |
|  | |
| HOUSING | |
| Adequate and healthy housing *(L. Farhang, 2008)* (9) | Preserve and construct housing in proportion to demand regarding size, affordability, and tenure. |
| Severe housing problems (%) *(D. Fastring, 2018)* (10) | Percentage of households with at least 1 of 4 housing problems: overcrowding, high housing costs, lack of kitchen facilities, or lack of plumbing facilities. |
| Overcrowded housing (%) *(A. Freitas, 2020)* (2) | Household overcrowding is an alternative measure of dwelling space that considers household composition. Dwelling space is an important dimension of housing quality. This indicator adopts the European Union agreed definition of overcrowding, which considers different needs for living space according to the age and gender composition of the household. A household is considered as living in overcrowded conditions if less than one room is available in each household: for each couple in the household; for each single person aged 18 or more; for each pair of people of the same gender between 12 and 17; for each single person between 12 and 17 not included in the previous category; and for each pair of children under age 12. Rooms refer to bedrooms, living and dining rooms and, in non-European countries, also kitchens. This indicator is calculated based on household surveys and measured as a percentage of all survey responses. |
| Ratio percentage of housing with more than 4 residents per housing mean area *(A. M. Novoa, 2018)* (7) | Ratio percentage of housing with more than 4 residents per housing mean area. |
| Households without central heating (%) *(A. Freitas, 2020)* (2) | Percentage of households without central heating. |
| Buildings without wheelchair access (%) *(A. Freitas, 2020)* (2) | Percentage of buildings without wheelchair access. |
| Older adults living in buildings with 3 floors or more without elevator (%) *(A. Freitas, 2020)* (2) | Percentage of older adults living in buildings with 3 floors or more without elevator. |
| Buildings in need of major repairs or very run-down (%) *(A. Freitas, 2020)* (2) | Percentage of buildings in need of major repairs or very run-down. |
| Housing value *(R. Mehdipanah, 2021)* (6) | Median housing value derived by homeowner’s estimates of their home value, including house and lot, mobile home, and lot or condominium unit. Although this does exclude housing values of renter properties, it provides an approximation that has been used to reflect neighbourhood wealth, quality, and affordability. |
| Home ownership (%) *(R. Mehdipanah, 2021)* (6) | Percentage of houses occupied by owners derived from the total number of owner-occupied houses occupied divided by the total number of houses occupied (renters and owners). |
| Occupied housing (%) *(R. Mehdipanah, 2021)* (6) | Percentage of occupied homes derived from the total number of occupied houses divided by all housing units (occupied and vacant). |
|  | |
| SANITATION | |
| Drinking water violations (yes/no) *(D. Fastring, 2018)* (10) | Indicator of the presence of health-related drinking water violations. 'Yes' indicates the presence of a violation, 'No' indicates no violation. Drinking Water Violations has only two values: Yes and No. A “Yes” indicates that at least one community water system in the county received at least one health-based violation during the specified period. A “No” indicates that there were no health-based drinking water violations in any community drinking water system in the county. |
| Proportion of households with drinking water supply (%) *(K. Makadzange, 2018)* (5) | Access to safe drinking water is measured by the percentage of the population having access to and using improved drinking water sources. |
| Increment in the wastewater collection network *(%) (K. Makadzange, 2018)* (5) | Access to sanitation is measured by the percentage of the population with access and using improved sanitation facilities. Improved sanitation facilities usually ensure separation of human excreta from human contact. |
|  | |
| INFRASTRUCTURE DOMAIN | |
| Public infrastructure/access to goods and services *(L. Farhang, 2008)* (9) | Ensure affordable and high-quality childcare for all neighbourhoods. Ensure accessible and high-quality educational facilities. Ensure accessible and high-quality educational facilities. Increase par, open space and recreation facilities. Ensure spaces for libraries, performing arts, theatre, museums, concerts, and festivals for personal and educational fulfillment. |
| Average walking distance to the nearest adult day-care centre (minutes) *(A. Freitas, 2020)* (2) | Average walking distance to the nearest adult day-care centre (minutes). |
| Average walking distance to the nearest sports facility (minutes) (*A. Freitas, 2020)* (2) | Average walking distance to the nearest sports facility (minutes). |
| Capacity of childcare centres (Number per 1000 children aged under 4) *(A. Freitas, 2020)* (2) | Capacity of childcare centres expressed as number per 1000 children aged under 4. |
| Neighbourhood area allocated to urban parks and gardens (%) *(A. M. Novoa, 2018)* (7) | Percentage of neighbourhood area allocated to urban parks and gardens. |
| Capacity of adult day-care centres (Number per 1000 population aged 65 years and over) *(A. Freitas, 2020)* (2) | Capacity of adult day-care centres expressed as number per 1000 population aged 65 years and over. |
|  | |
| ENVIRONMENT | |
| Environmental stewardship *(L. Farhang, 2008)* (9) | Decrease consumption of energy and natural resources. Restore, preserve, and protect healthy natural habitats. Promote food access and sustainable urban and rural agriculture. Promote productive reuse of previously contaminated sites. Preserve clean air quality. Maintain safe levels of community noise. |
| Air pollution *(D. Fastring, 2018)* (10) | Air Pollution measures the particulate matter in the air. It reports the average daily density of fine particulate matter in micrograms per cubic meter. Fine particulate matter is defined as particles of air pollutants with an aerodynamic diameter less than 2.5 micrometers (PM2.5). |
| Particulate matter (PM10) concentrations (µg/m3) *(A. Freitas, 2020; R. Mehdipanah, 2021)* (2,6) | PM10 is the concentration of particles with a diameter equal to or greater than 10 microns (μ), which are usually produced from construction and mechanical activities. WHO has set guidelines for PM10 at 20 μg/m3 annual mean. Diesel PM values were derived based on PM10 emissions from on-road and non-road mobile sources burning diesel or residual fuels. The exposure measure consisted of estimated inhalation exposure concentrations of diesel PM modelled based on annual average ambient outdoor concentration, human activity patterns, demographic features, and micro environmental factors. |
| Premature mortality attributed to PM2.5 concentrations *(K.Oliveira, 2022)* (11) | Increase in the risk of mortality of 6.2% in adults age 30 and over for a 10 μg/m3 increase of PM2.5. |
| Premature mortality attributed to PM2.5 concentrations *(K.Oliveira, 2022)* (11) | Increase in the risk of mortality of 4% in adults age 30 and over for a 10 μg/m3 increase of PM10. |
| Premature mortality attributed to NO_2_ concentrations *(K.Oliveira, 2022)* (11) | Increase in the risk of mortality of 5.5% in adults age 30 and over for a 10 μg/m3 increase of PM10. |
| Population exposed to noise levels greater than Lden55 dB (%) *(A. Freitas, 2020)* (2) | Percentage of population exposed to noise levels greater than Lden55 db. |
| Population potentially affected by flooding (%) *(A. Freitas, 2020)* (2) | Percentage of population potentially affected by flooding. |
| Vegetation index *(A. M. Novoa, 2018)* (7) | Indicator that reflects the amount of vegetation by assessing the amount of green observed from a satellite picture. |
|  | |
| EDUCATION | |
| School drop-out rate (%) *(A. Freitas, 2020)* (2) | School dropout rate. |
| Number of children enrolled in primary education *(L. O. Andrade, 2005)* (3) | Number of children enrolled in primary education. |
| Literacy rate of children in the first cycle of primary education *(L. O. Andrade, 2005)* (3) | Literacy rate of children in the first cycle of primary education. |
| People 16 to 29 years with primary level education or less (%) *(A. M. Novoa, 2018)* (7) | Percentage of people 16 to 29 years with primary level education or less. |
| High school education (%) *(R. Mehdipanah, 2021)* (6) | Percentage with high school diploma was derived from the total number of individuals with a high school diploma divided by the total population. |
| Bachelor's degree (%) *(R. Mehdipanah, 2021)* (6) | Percentage with bachelor’s degree or more was derived from the total number of individuals with a bachelor’s degree or more, by the total population. |
|  | |
| ECONOMIC CONDITIONS AND SOCIAL PROTECTION | |
| Healthy economy *(L. Farhang, 2008)* (9) | Increase high-quality employment opportunities for local residents. Increase jobs that provide healthy, safe, and meaningful work. Increase equality in income and wealth. Benefits and protects natural resources and the environment. |
| Family available income index *(A. M. Novoa, 2018)* (7) | Compound index that reflects the distribution of the neighbourhood mean family income compared to the city mean and that includes 5 indicators (people aged 25 years or more with university level education (%), registered unemployment among people aged 16 to 64 years (%), number of cars per inhabitant, new cars (less than 2 years) with more than 16 hp (%) and second-hand housing prices). |
| Unemployment rate (%) *(A. Freitas, 2020)* (2) | This indicator is measured in numbers of unemployed people as a percentage of the labour force, and it is seasonally adjusted. The labour force is defined as the total number of unemployed people plus those in employment. |
| Employment rate (%) *(R. Mehdipanah, 2021)* (6) | Percentage employed was derived from the total number of employed individuals divided by the total population in the labour force (employed and unemployed). |
| Registered unemployment among people aged 16 to 64 years (%) *(A. M. Novoa, 2018)* (7) | One of the 5 indicators belonging to family available income index. |
| Youth neither employed nor in education or training (%) *(A. Freitas, 2020)* (2) | Percentage of youth neither employed nor in education or training. |
| Homeless people (number) *(A. Freitas, 2020)* (2) | Number of homeless people. |
| People receiving social integration subsidies (number per 1000 active population) *(A. Freitas, 2020)* (2) | People receiving social integration subsidies expressed as number per 1000 active population. |
| Rate of people aged 17 years and younger assisted by the child and teenage assistance team *(A. M. Novoa, 2018)* (7) | Rate of people aged 17 years and younger assisted by the child and teenage assistance team. |
| Rate of people assisted by social services excluding those attributable to the dependency law *(A. M. Novoa, 2018)* (7) | Rate of people assisted by social services excluding those attributable to the dependency law. |
| Children living above the poverty line (%) *(R. Mehdipanah, 2021)* (6) | Nonpoverty status was determined by comparing the total family income with the poverty threshold relative to the family size and composition. Percentage of children living above poverty line was derived from the total number of children not in poverty divided by total of households with children. |

## **Table S3. PRISMA Checklist**

| **Section and Topic** | **Item #** | **Checklist item** | **Location where item is reported** |
| --- | --- | --- | --- |
| **TITLE** | | |  |
| Title | 1 | Identify the report as a systematic review. | Title |
| **ABSTRACT** | | |  |
| Abstract | 2 | See the PRISMA 2020 for Abstracts checklist. | Abstract |
| **INTRODUCTION** | | |  |
| Rationale | 3 | Describe the rationale for the review in the context of existing knowledge. | Introduction |
| Objectives | 4 | Provide an explicit statement of the objective(s) or question(s) the review addresses. | Introduction |
| **METHODS** | | |  |
| Eligibility criteria | 5 | Specify the inclusion and exclusion criteria for the review and how studies were grouped for the syntheses. | Methods – Selection criteria |
| Information sources | 6 | Specify all databases, registers, websites, organisations, reference lists and other sources searched or consulted to identify studies. Specify the date when each source was last searched or consulted. | Methods – Search strategy  Supplementary material – Section S1 |
| Search strategy | 7 | Present the full search strategies for all databases, registers, and websites, including any filters and limits used. | Supplementary material – Section S1 |
| Selection process | 8 | Specify the methods used to decide whether a study met the inclusion criteria of the review, including how many reviewers screened each record and each report retrieved, whether they worked independently, and if applicable, details of automation tools used in the process. | Methods – Selection criteria |
| Data collection process | 9 | Specify the methods used to collect data from reports, including how many reviewers collected data from each report, whether they worked independently, any processes for obtaining or confirming data from study investigators, and if applicable, details of automation tools used in the process. | Methods – Selection criteria |
| Data items | 10a | List and define all outcomes for which data were sought. Specify whether all results that were compatible with each outcome domain in each study were sought (e.g. for all measures, time points, analyses), and if not, the methods used to decide which results to collect. | Methods – Data extraction and analysis |
|  | 10b | List and define all other variables for which data were sought (e.g. participant and intervention characteristics, funding sources). Describe any assumptions made about any missing or unclear information. | Methods – Data extraction and analysis |
| Study risk of bias assessment | 11 | Specify the methods used to assess risk of bias in the included studies, including details of the tool(s) used, how many reviewers assessed each study and whether they worked independently, and if applicable, details of automation tools used in the process. | NA |
| Effect measures | 12 | Specify for each outcome the effect measure(s) (e.g. risk ratio, mean difference) used in the synthesis or presentation of results. | NA |
| Synthesis methods | 13a | Describe the processes used to decide which studies were eligible for each synthesis (e.g. tabulating the study intervention characteristics and comparing against the planned groups for each synthesis (item #5)). | Methods – Data extraction and analysis  Table 2, Table 3, Table S2 |
|  | 13b | Describe any methods required to prepare the data for presentation or synthesis, such as handling of missing summary statistics, or data conversions. | NA |
|  | 13c | Describe any methods used to tabulate or visually display results of individual studies and syntheses. | Results  Table 1, Table 2, Table 3, Table S2  Figure S1, Figure S2 |
|  | 13d | Describe any methods used to synthesize results and provide a rationale for the choice(s). If meta-analysis was performed, describe the model(s), method(s) to identify the presence and extent of statistical heterogeneity, and software package(s) used. | Methods – Data extraction and analysis |
|  | 13e | Describe any methods used to explore possible causes of heterogeneity among study results (e.g. subgroup analysis, meta-regression). | NA |
|  | 13f | Describe any sensitivity analyses conducted to assess robustness of the synthesized results. | NA |
| Reporting bias assessment | 14 | Describe any methods used to assess risk of bias due to missing results in a synthesis (arising from reporting biases). | NA |
| Certainty assessment | 15 | Describe any methods used to assess certainty (or confidence) in the body of evidence for an outcome. | NA |
| **RESULTS** | | |  |
| Study selection | 16a | Describe the results of the search and selection process, from the number of records identified in the search to the number of studies included in the review, ideally using a flow diagram. | Results  Figure 1 |
|  | 16b | Cite studies that might appear to meet the inclusion criteria, but which were excluded, and explain why they were excluded. | Supplementary material – Section S2 |
| Study characteristics | 17 | Cite each included study and present its characteristics. | Results  Table 1 |
| Risk of bias in studies | 18 | Present assessments of risk of bias for each included study. | NA |
| Results of individual studies | 19 | For all outcomes, present, for each study: (a) summary statistics for each group (where appropriate) and (b) an effect estimate and its precision (e.g. confidence/credible interval), ideally using structured tables or plots. | Results  Table 1, Table 2, Table S2 |
| Results of syntheses | 20a | For each synthesis, briefly summarise the characteristics and risk of bias among contributing studies. | NA |
|  | 20b | Present results of all statistical syntheses conducted. If meta-analysis was done, present for each the summary estimate and its precision (e.g. confidence/credible interval) and measures of statistical heterogeneity. If comparing groups, describe the direction of the effect. | NA |
|  | 20c | Present results of all investigations of possible causes of heterogeneity among study results. | NA |
|  | 20d | Present results of all sensitivity analyses conducted to assess the robustness of the synthesized results. | NA |
| Reporting biases | 21 | Present assessments of risk of bias due to missing results (arising from reporting biases) for each synthesis assessed. | NA |
| Certainty of evidence | 22 | Present assessments of certainty (or confidence) in the body of evidence for each outcome assessed. | NA |
| **DISCUSSION** | | |  |
| Discussion | 23a | Provide a general interpretation of the results in the context of other evidence. | Discussion – Paragraph 1 |
|  | 23b | Discuss any limitations of the evidence included in the review. | Discussion – Paragraph 5 |
|  | 23c | Discuss any limitations of the review processes used. | Discussion – Paragraph 5 |
|  | 23d | Discuss implications of the results for practice, policy, and future research. | Discussion – Paragraph 6 |
| **OTHER INFORMATION** | | |  |
| Registration and protocol | 24a | Provide registration information for the review, including register name and registration number, or state that the review was not registered. | Methods – Registration and protocol deviations |
|  | 24b | Indicate where the review protocol can be accessed, or state that a protocol was not prepared. | Methods – Registration and protocol deviations |
|  | 24c | Describe and explain any amendments to information provided at registration or in the protocol. | NA |
| Support | 25 | Describe sources of financial or non-financial support for the review, and the role of the funders or sponsors in the review. | Title page |
| Competing interests | 26 | Declare any competing interests of review authors. | Title page |
| Availability of data, code and other materials | 27 | Report which of the following are publicly available and where they can be found: template data collection forms; data extracted from included studies; data used for all analyses; analytic code; any other materials used in the review. | Available under request |

# Supplemental references

1. Baum F, Cooke R. Healthy cities Australia: The evaluation of the pilot project in Noarlunga, South Australia. Health Promotion International. 1992;7(3).

2. Freitas Â, Rodrigues TC, Santana P. Assessing Urban Health Inequities through a Multidimensional and Participatory Framework: Evidence from the EURO-HEALTHY Project. Journal of Urban Health. 2020;97(6).

3. Andrade LOM, Bareta IC de HC, Gomes CF, Canuto OMC. Public health policies as guides for local public policies: the experience of Sobral-Ceará, Brazil. Promot Educ. 2005;Suppl 3.

4. Junqueira V, Pessoto UC, Kayano J, Nascimento PR, Castro IE do N, Rocha JL da, et al. Equity in the health sector: evaluation of public policy in Belo Horizonte, Minas Gerais State, Brazil, 1993-1997. Cadernos de saúde pública / Ministério da Saúde, Fundação Oswaldo Cruz, Escola Nacional de Saúde Pública. 2002;18(4).

5. Makadzange K, Radebe Z, Maseko N, Lukhele V, Masuku S, Fakudze G, et al. Implementation of Urban Health Equity Assessment and Response Tool: a Case of Matsapha, Swaziland. Journal of Urban Health. 2018;95(5).

6. Mehdipanah R, Israel BA, Richman A, Allen A, Rowe Z, Gamboa C, et al. Urban HEART Detroit: the Application of a Health Equity Assessment Tool. Journal of Urban Health. 2021;98(1).

7. Novoa AM, Pérez G, Espelt A, Echave C, de Olalla PG, Calvo MJ, et al. The Experience of Implementing Urban HEART Barcelona: a Tool for Action. Journal of Urban Health. 2018;95(5).

8. Riley C, Roy B, Lam V, Lawson K, Nakano L, Sun J, et al. Can a collective-impact initiative improve well-being in three US communities? Findings from a prospective repeated cross-sectional study. BMJ Open. 2021;11(12):e048378.

9. Farhang L, Bhatia R, Scully CC, Corburn J, Gaydos M, Malekafzali S. Creating tools for healthy development: Case study of San Francisco’s eastern neighborhoods community health impact assessment. Vol. 14, Journal of Public Health Management and Practice. 2008.

10. Fastring D, Mayfield-Johnson S, Funchess T, Egressy J, Wilson G. Investing in gulfport: Development of an academic–community partnership to address health disparities. Progress in Community Health Partnerships: Research, Education, and Action. 2018;12(Special Issue).

11. Oliveira K, Rodrigues V, Slingerland S, Vanherle K, Soares J, Rafael S, et al. Assessing the impacts of citizen-led policies on emissions, air quality and health. J Environ Manage. 2022;302(Pt A):114047.
